# Lipid Profiling of Pacific Abalone (*Haliotis discus hannai*) at Different Developmental Stages Using Ultrahigh Performance Liquid Chromatography-Tandem Mass Spectrometry

**DOI:** 10.1155/2022/5822562

**Published:** 2022-10-17

**Authors:** Hey Gene Lee, MinJoong Joo, Jong-Moon Park, Mi Ae Kim, JeongHun Mok, Seong-Hyeon Cho, Young Chang Sohn, Hookeun Lee

**Affiliations:** ^1^College of Pharmacy, Gachon University, Incheon 21936, Republic of Korea; ^2^Basilbiotech, Incheon 22002, Republic of Korea; ^3^Department of Marine Molecular Bioscience, Gangneung-Wonju National University, Gangneung 25457, Republic of Korea; ^4^East Coast Life Sciences Institute, Gangneung-Wonju National University, Gangneung 25457, Republic of Korea

## Abstract

Pacific abalone (*Haliotis discus hannai*) is a commercially important mollusk; therefore, improvement of its growth performance and quality has been emphasized. During embryonic development, abalones undergo a series of distinct larval stages, including swimming veliger larvae, juveniles, and mature individuals, and their biomolecular composition varies depending on the developmental stage. Therefore, in the present study, we performed untargeted lipid profiling of abalone tissues at different developmental stages as well as the hemolymph of mature female and male abalones using ultrahigh-performance liquid chromatography-tandem mass spectrometry. These profiles can provide meaningful information to understand compositional changes in lipids through abalone metamorphosis and development. A total of 132 lipids belonging to 15 classes were identified from abalone tissues at different developmental stages. Moreover, 21 lipids belonging to 8 classes were identified from the hemolymph of mature abalones. All data were processed following strict criteria to provide accurate information. Triglycerides and phosphatidylcholines were the major lipid components identified in both tissues and hemolymph, accounting for, respectively, 27% and 15% of all lipids in tissues and, respectively, 24% and 38% of all lipids in the hemolymph. Of note, lysophosphatidylcholine was only detected in the tissues of mature abalones, paving the way for further analyses of abalone lipids based on developmental stages. The present findings offer novel insights into the lipidome of abalone tissues and hemolymph at different developmental stages, building a foundation for improving the efficiency and quality of abalone aquaculture.

## 1. Introduction

Abalones are large marine gastropods widely distributed in coastal waters worldwide [1]. These mollusks are well-known for their nutritional benefits and are widely consumed in East Asia, including South Korea and China [2, 3]. Among the various abalone species, Pacific abalone (*Haliotis discus hannai*) is commercially important [4, 5]. Therefore, substantial efforts have been devoted to improve its quality, and research on the embryology of its aquaculture is paramount [6]. This gastropod undergoes a series of life cycle stages, including the pelagic and benthic phases [7]. The pelagic phase marks a free-living period and includes trochophores, swimming larvae, and swimming veliger larvae. Subsequently, the larvae undergo morphological changes to enter the benthic phase, initiating sedentary life and undergoing further metamorphosis to turn into postlarvae. Thereafter, they morph into a young shell or a juvenile abalone, and finally, grow into a sexually mature abalone [8–10]. Mature abalones reach their marketable size in approximately 4 years, which is relatively long [11]. Meanwhile, given the substantial expansion of global abalone aquaculture [12], improvement of the nutritive quality of these mollusks within a short period is imperative [13].

To achieve the above goal, molecular mechanisms underlying abalone growth and development must be elucidated for sustainable aquaculture management [14]. Over the past few decades, abalone development, growth, reproduction, and adaptation have been extensively studied [15–17]. These efforts were aimed at improving the growth performance, efficiency, and qualitative aspects of abalones. Furthermore, abalones have been studied through various omics technologies, including transcriptomics [18–20], genomics [21–23], proteomics [8, 24], and metabolomics [16, 25–27].

Nonetheless, the biomolecular coverage of the commercially important Pacific abalone (*H. discus hannai*) remains limited [15]. Genomic and transcriptomic analyses have been used to identify meaningful markers for abalone growth [19, 20, 22, 28–30]. A transcriptomic study of sexual maturation in *H. discus hannai* has uncovered certain pathways related to maturation, with lipid pathways being crucial in this process [19]. In addition, genomic studies aimed at the identification of growth-related single-nucleotide polymorphisms (SNPs) in *H. discus hannai* have revealed certain lipids involved in abalone growth and proposed genes encoding enzymes that are related to the metabolism of these lipids (e.g., mitochondrial glycerol-3-phosphate acyltransferase1, *gpam*) as reliable candidate markers for growth [22, 31].

However, detailed mechanisms that orchestrate the entire developmental process remain to be elucidated, and systematic identification of lipids involved in this process has not been performed in abalone species. Abalone comprises many components, including water, proteins, salts, carbohydrates, and lipids [13]. Although lipids are present in a low proportion, they are the key components of abalone, in that they are part of various biological systems (e.g., membrane) and processes (e.g., development and cellular signaling) of organisms [32, 33]. Furthermore, lipids are essential for optimal growth in mollusk species [34]. Given its importance, the lipid composition of abalone tissues has been studied to improve growth and nutritional aspects.

The key factors of lipidomics include lipid coverage, sensitivity, identification, and throughput. Thanks to recent advances in mass spectrometry (MS) and high-efficiency separation techniques, rapid and sensitive detection of a large number of individual lipids in various biological samples is possible [35]. The lipid profiles of two abalone species have been determined using gas chromatography-mass spectrometry (GC-MS) [13]. In addition, lipids in different tissues of *H. discus hannai* have been identified using liquid chromatography-mass spectrometry (LC-MS) [33]. To date, however, there has been no study on lipidomics at a different developmental stage of abalone.

Here, we explored the synthesis, regulation, and accumulation of lipids through lipid profiling at different developmental stages of *H. discus hannai*. The knowledge of lipid composition and its changes during development will be important to understand abalone development. Additionally, lipid profiling of the hemolymph of mature females and males will provide meaningful data on lipid homeostasis and abalone maturation.

To this end, in the present study, abalone lipid profiles were analyzed using ultrahigh-performance liquid chromatography-tandem mass spectrometry (UHPLC-MS/MS) through a nontargeted approach. The objective of the study was to obtain detailed lipid profiles of abalone tissues at different developmental stages and hemolymph of mature females and males. This information will be valuable for improving the growth performance and commercial value of abalone aquaculture.

## 2. Materials and Methods

### 2.1. Data Processing and Statistical Analysis

*Haliotis discus hannai* was purchased from a local market in Gangneung, Gangwon-do Province, South Korea, and mature individuals were selected. For lipidomics, the cerebral ganglia (CG), pleuropedal ganglion mass (PPG), ovaries (O), testes (T), hepatopancreas (HP), gills (G), intestines (I), eyes (E), cephalic tentacles (CT), epipodium tentacles (ET) and feeding organs (F) were dissected from three mature females (body weight (BW) 93.82 ± 3.59 g; shell length (SL), 93.3 ± 2.9 mm) and three mature males (BW, 74.09 ± 13.44 g; SL, 86.7 ± 5.8 mm). Then, all dissected female and male tissues were pooled (CG + PPG + O + T + HP + G + I + E + CT + ET + F).

Artificial fertilization was performed as described previously [36]. Sexually mature *H. discus hannai* (approximately 5 years old, with a mean SL of 120 mm) individuals were induced to release eggs and sperm using the conventional method of air exposure, followed by ultraviolet (UV)-irradiated seawater treatment. The eggs and sperm were thoroughly mixed to minimize individual variability. Following fertilization, the developing embryos were cultured in filtered and aerated seawater at 17°C. Two groups of *H. discus hannai* larvae at different developmental stages, including the late veliger stage (4 days postfertilization, dpf) larvae (also called swimming veliger larvae), identified microscopically, and the juvenile stage (averaging ∼4 mm in SL, 90 dpf), were collected. All samples were stored in a deep freezer at −80°C to keep them fresh.

### 2.2. Chemicals

HPLC-grade methanol, water, acetonitrile, and 2-propanol were purchased from JT Baker (Philipsburg, NJ, USA). HPLC-grade formic acid was purchased from Fluka, Sigma-Aldrich (St. Louis, MO, USA). Chloroform, ammonium formate, and hydrochloric acid were purchased from Sigma-Aldrich (St. Louis, MO, USA).

### 2.3. Experimental Design

#### 2.3.1. Lipid Extraction from Tissues

Before lipid extraction, fresh frozen abalone tissues in liquid nitrogen were individually pulverized using a Covaris CP02 cyro-prep instrument (Covaris, Woburn, USA). For lipid extraction from tissues, a two-step method involving neutral and acidic extraction was used. First, in neutral extraction, 1 mL of chloroform:methanol (1 : 2, v/v) was added to the cell pellets [37]. The samples were incubated for 10 min on ice and centrifuged at 13,800 × g for 2 min at 4°C. Then, 950 *μ*L of the supernatant was transferred to a fresh clean tube. Next, in acidic extraction, 750 *μ*L of chloroform:methanol:HCl (1N, 37%) (40 : 80 : 1, v/v/v) was added to the remaining samples. After incubating for 15 min at room temperature, 250 *μ*L of cold chloroform and 450 *μ*L of cold 0.1 M HCl were added to the sample. The mixture was vortexed for 1 min and centrifuged at 6,500 × g for 2 min at 4°C. The lower organic phase was collected and combined with the prior extract. The sample was then dried using the Scan Speed 40 centrifugal evaporator (1,800 rpm, 3 h; Labogene, Denmark). Before starting the lipid analysis, the sample was dissolved in 50 *μ*L of mobile phase solvent A:solvent B (2 : 1, v/v).

#### 2.3.2. Lipid Extraction from Hemolymph

**Figure 1:**
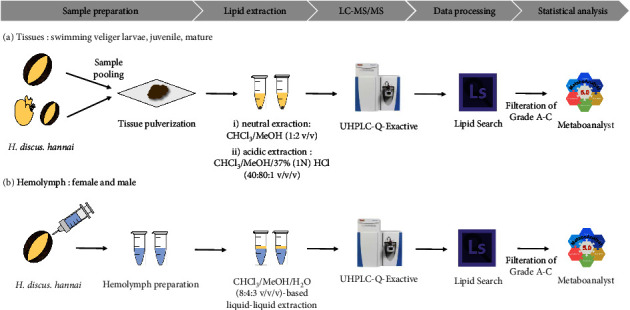
Workflow of the profile profiling of (a) abalone (*Haliotis discus hannai*) tissues at different development stages (swimming veliger larvae, juveniles, and mature adults) and (b) the hemolymph of female and male abalones.

Lipids from the hemolymph samples were extracted according to the Folch method [38] using a mixture of chloroform and methanol (2 : 1, v/v). The samples were vortexed and incubated on ice for 30 min. After the addition of chloroform:methanol:water (8 : 4:3, v/v/v), the samples were incubated on ice for an additional 10 min and centrifuged at low speed (2,000 rpm) for 5 min at 4°C. The lower organic phase was transferred to a fresh tube, and the aqueous layer was re-extracted with 1 mL of chloroform: methanol (2 : 1, v/v). The organic phases were combined and dried using the Scan Speed 40 centrifugal evaporator (1,800 rpm, 3 h; Labogene). The overall experimental workflow is presented in Figure 1.

### 2.4. LC-MS/MS Analysis

Untargeted UHPLC-MS/MS analyses were performed on the Agilent 1290 Infinity UHPLC system (Agilent Technologies, Wilmington, DE) coupled to the Q-Exactive Orbitrap mass spectrometer (Thermo Fischer Scientific, Waltham, MA, USA) with heated electrospray ionization (HESI). Chromatographic separation was achieved using the Hypersil GOLD™ C18 HPLC column (2.1 × 100 mm; 1.9 *μ*m particle size; Thermo Fischer Scientific) maintained at 25°C. The mobile phases used were solvent A (acetonitrile:methanol:water (19 : 19 : 2, v/v/v] + 20 mmol L^−1^ ammonium formate + 0.1% [v/v] formic acid) and solvent B (2-propanol + 20 mmol L^−1^ ammonium formate + 0.1% [v/v] formic acid). Lipids were separated using a gradient elution program as follows: 0–5 min, 5% B; 5–15 min, 5–30% B; 15–22 min, 30–90% B; 22–25 min, 90% B; 25–26 min, 90–5% B; 26–30 min, 5% B. The flow rate was 150 *μ*L·min^−1^, and the total run time was 30 min. The injection volume was 5 *μ*L per run. Mass spectrometric detection was performed through HESI in the positive mode, with a full scan range of *m*/*z* 150 to 2,000. The resolution of the spectrometer was set at 70,000. The HESI parameters were set as follows: sheath gas flow rate, 5 arb; auxiliary gas flow rate, 5 arb; sweep gas flow rate, 0; spray voltage, 4.0 kV; capillary temperature, 320°C; and S-lens radio frequency (RF), 50%. All samples were analyzed in technical triplicate.

### 2.5. Data Processing and Statistical Analysis

MS data were acquired and processed using Xcalibur (v4. 1. 31.9, Thermo Fischer Scientific, San Jose, CA, USA). The obtained raw data were uploaded and processed using Lipid Search 5.0 (Thermo Fischer Scientific, San Jose, CA, USA) for lipid profiling under the following conditions: product search; 5.0 ppm precursor ion mass tolerance; 8.0 ppm product ion tolerance; 1.0% intensity threshold; and top rank, main isomer peak, and FA priority filters. To filter inaccurate identification, lipids that were graded A, B and C were selected [39, 40]. In addition, lipids with odd-numbered fatty acid chains, commonly called bacterial fatty acids, were filtered out to accurately consider animal lipids [41–43]. Lipids with % rsd values exceeding 50 were also removed to minimize error [44, 45]. For statistical analysis, Metabo Analyst 5.0 (https://www.metaboanalyst.ca/) was used [46]. Multivariate statistical analyses, including principal component analysis (PCA), partial least squares-discriminant analysis (PLS-DA), and heat map clustering, were used.

## 3. Results and Discussion

### 3.1. Overview of *H. discus hannai* Lipid Profiling

To achieve reproducible sample extraction, liquid nitrogen was used to homogenize the tissue of abalone. Different modified extraction methods based on chloroform and methanol were used to extract lipids from tissue and hemolymph, for increased coverage of lipid species in the sample [47]. Optimized mobile phases A and B were used for efficient ionization and separation in liquid chromatography. Each lipid class was well separated showing identical elution tendency as the previous research results on lipid separation in LC [47, 48].

For data processing, rather than using the entire data set, we applied strict criteria and filtered the data to ensure high reliability. Only features that can confirm MS^2^ data were used. As shown in Table S1, the ppm value for all lipid species was <0.01.

Additionally, lipid species containing odd-numbered fatty acids have been identified in several previous studies. In the present study, such lipid species were removed, as they are not common because of fatty acid metabolism and synthesis.

In this context, the following are the strengths of the present study: (1) To homogenize the tissues and efficiently extract the lipids, cyro-prep using liquid nitrogen followed for extraction in the first step. (2) Instead of using GC-MS, which can identify only a few central lipids, UHPLC-Quadrupole-Orbitrap-MS was used for scan-type lipidomics, which is appropriate for the analysis of phospholipids, neutral lipids, or sphingolipids [47]. (3) Greater coverage of lipidome was attained using cutting-edge software (Lipidsearch 5.0) and a comprehensive database. (4) A filter for lipids with odd-numbered fatty acids was added during data processing.

### 3.2. Lipid Profiling of Abalone Tissue

**Figure 2:**
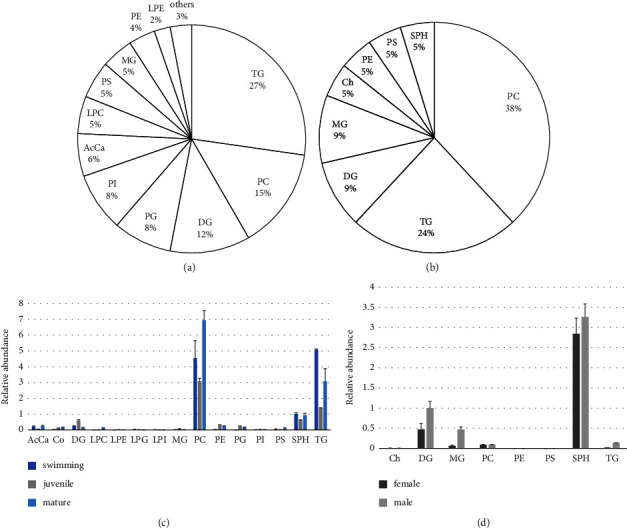
Lipid class composition of abalone. (a) Relative lipid composition of total abalone tissues. (b) Relative lipid composition of total abalone hemolymph. (c) Relative abundance of the normalized average peak area of lipid classes in abalone tissues at three different developmental stages (swimming veliger larvae, juvenile, and mature adults). (d) Relative abundance of the normalized average peak area of lipid classes in the hemolymph of female and male abalones.

To observe the overall changes in lipid composition and distribution during *H. discus hannai* development, we extracted lipids from swimming veliger larvae, juveniles, and mature abalones and subjected these to LC-MS/MS analysis. Finally, 132 lipids were identified in *H. discus hannai*: 36 triglyceride (TG) types (27.3%); 19 phosphatidylcholine (PC) types (14.4%); 15 diglyceride (DG) types (15%); 11 phosphatidylglycerol (PG) types (8.3%); 11 phosphatidylinositol (PI) types (8.3%); 8 acylcarnitine (AcCa) types (6.1%); 7 lysophosphatidylcholine (LPC) types (6.1%); 7 phosphatidylserine (PS) types (6.1%); 6 monoglyceride (MG) types (4.5%); 5 phosphatidylethanolamine (PE) types (3.8%); 3 lysophosphatidylethanolamine (LPE) types (2.3%) and other lipid types, including coenzyme (Co), lysophosphatidylglycerol (LPG), lysophosphatidylinositol (LPI), and sphinganine (SPH) (Figure 2(a)). Total ion current (TIC) chromatograms of tissue samples are provided in Figure S1 and detailed data (Rt, *m*/*z*, ppm, adduct ion, area, and % rsd) for each of the 132 lipid species are presented in Table S1. TGs, DGs, PGs, PIs, and Co were detected as [M + NH_4_]^+^ adducts, while the remaining lipids were identified in the protonated form [M + H]^+^. Our results are consistent with lipid adduct ions identified in previous studies [49]. The [M + NH_4_]^+^ ion was likely produced by ammonium formate in the LC mobile phase. The [M + Na]^+^ and [M + K]^+^ ions are produced during the extraction process, HPLC valve operation, or ESI; however, based on the previous data [33], this was judged as noise and not considered for lipid identification. The highest number of TG types was eluted between 17 and 25 min, and the highest number of PC types was between 6 and 20 min. Therefore, the longer the alkyl chain and saturated lipid, the longer the retention time. This result is consistent with previous reports on abalone lipidomics, in which TGs accounted for a high proportion among lipid classes; as such, these TGs may be derived from the viscera or gonads, rather than foot tissues [50]. The relative abundance of the normalized average peak area of lipid classes in abalone tissues according to the developmental stage is represented in Figure 2(c) and the list of normalized peak areas of each lipid species is shown in Table S4. A total of 15 classes were identified, and PCs and TGs accounted for the highest proportion of lipids at all stages. In juveniles, most lipid species were present at lower proportions than in swimming and mature individuals, except for DG, which was nearly two times more abundant in juveniles. Interestingly, one characteristic feature of the intersample lipid profile is that LPCs were only detected in mature individuals (Table S1) [51]. LPCs were eluted between 6 and 20 min and were detected as [M + H]^+^ adducts. Lysophospholipids, as intermediates of glyceride metabolism, act as mediators in several neural pathways [52]. Lysophospholipids are produced by the action of phospholipase (PL)A_1_ and PLA_2_ and are either hydrolyzed by lysophospholipase or used to regenerate phospholipids through the remodeling pathway [53]. In the remodeling pathway of phospholipid synthesis, lysophospholipids are transiently produced by the action of PLA_2_ but rapidly acylated to acyl-CoA through the diacylation reaction cycle for the maintenance of normal and essential neuronal membrane composition [54]. A previous study on the transcriptome of *H. discus hannai* demonstrated that the MtsPLA_2_ gene related to PLA_2_ was highly expressed in mature abalone [19]. In the present study, as LPCs were detected only in mature abalones, additional attention should be paid to the dynamics of these lipids during the development of this mollusk in the future. Specifically, integrated genomic, transcriptomic, and proteomic analyses are warranted to identify, among the profiled lipid species, useable candidates for the rapid growth and successful aquaculture of abalones.

### 3.3. Lipid Profiling of Mature Abalone Hemolymph

To the best of our knowledge, the present study is the first to analyze lipid profiles of the hemolymph of *H. discus hannai* [13, 33, 55]. Information on the lipid profiles of the hemolymph of aquatic mollusks is limited to TGs and cholesterol in the studies of *Pomacea canaliculata* [56], *Mercenaria mercenaria* [57], and *Achatina fulica* [58]. We performed LC-MS/MS analysis of lipids extracted from the hemolymph of mature female and male abalones and identified a total of 21 lipids belonging to 8 classes (note that this refers to the sum of lipids from both female and male abalones). 8 PC types (38.1%), accounted for the highest proportion of all lipids, followed by 5 TG types (23.8%), 2 DG types (9.5%), and 2 MG types (9.5%). Cholesterol, PE, PS, and SPH types were identified in only one sample, accounting for 5% of the total lipids (Figure 2(b)). Overall, the observed lipid profile of the hemolymph of abalone, characterized by abundant PCs and TGs, is similar to that of other living blood samples [59]. TIC chromatograms of hemolymph samples are provided in Figure S2 and detailed data (Rt, *m*/*z*, ppm, adduct ion, area, and % rsd) of each of the 21 lipid species are presented in Table S2. TGs and DGs were detected as [M + NH_4_]^+^ adducts and cholesterol was identified as a dehydrated protonated molecule ([M + H–H_2_O]^+^) [60, 61]. In hemolymph analysis, [M + Na]^+^ and [M + K]^+^ ions were considered background noise, similar to that in tissue analysis. The relative percentage of the normalized average peak area of lipid classes in the hemolymph of female and male abalones is shown in Figure 2(d) and the list of normalized peak areas of each lipid species is shown in Table S4. Compared with tissue, the hemolymph exhibited a higher proportion of SPHs. Specifically, SPH was the most abundant lipid in hemolymph, and its proportion was over three times the proportion of DG, which was the second most abundant lipid in the hemolymph. SPH is involved in the regulation of various cellular functions, including cell growth, and constitutes part of the lipoprotein particles circulating in animal blood [62]. The percentage of peak area of DGs was followed by that of PCs and TGs, and their levels were comparable to those recorded in the human blood [63]. Although PCs and TGs were the most diverse species, they were relatively less abundant, and this result is contrary to the finding that these are the major lipids in human blood [59]. Lipid species abundance differed between females and males. While PE and PS were identified only in males, their average peak area was relatively small, indicating no significant differences.

### 3.4. Detailed Characterization of Altered Lipids of Each Group in Tissue and Hemolymph

**Figure 3:**
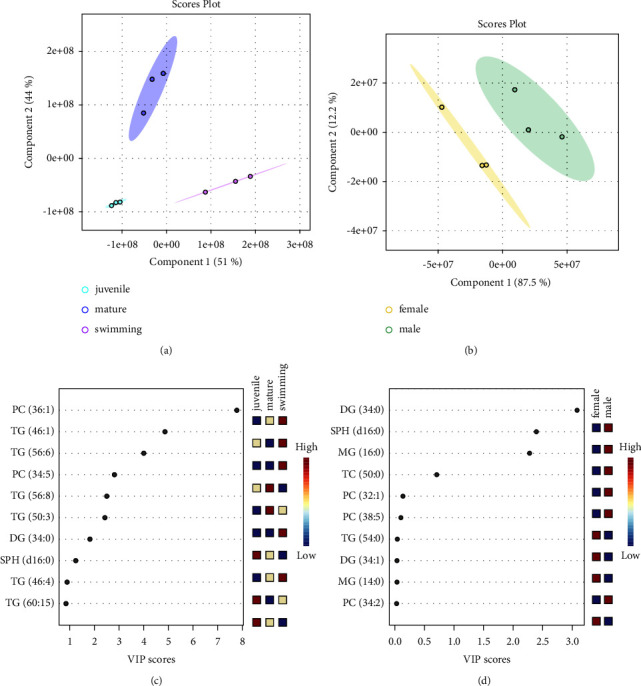
Partial least squares-discriminant analysis (PLS-DA) score plots and variable importance in projection (VIP) score plots displaying the top 10 most important lipids in abalone tissues and hemolymph. (a) PLS-DA score plot of tissues at three different developmental stages. (b) PLS-DA score plot of female and male hemolymph. In the PLS-DA score plots, the colored ellipse represents the 95% confidence interval. (c) Top 10 lipids with the highest VIP scores in tissues at three different developmental stages. (d) Top 10 lipids with the highest VIP scores in female and male hemolymph.

Intratissue and intertissue sample variations were visualized using PCA, as shown in Figure S3(a), which revealed marked differences among the three groups. Juvenile samples were grouped into the most compact clusters, with the smallest 95% confidence interval. PC1 explained 54.8% of the variance, while PC2 explained 40.7% of the variance. Each sample group was clearly separated. Furthermore, PLS-DA, which is a supervised statistical method [64], was applied for the classification of the three groups, as shown in Figure 3(a). Similar to that in the PCA biplot, juvenile samples formed a more compact cluster, and each group was clearly separated, indicating distinct differences in lipid profiles among the groups. In the PLS-DA score plot, components 1 and 2 explained, respectively, 51% and 44% of the total variance.

Among the 132 lipids, the VIP score plot of the top 10 lipids selected based on the PLS-DA model is shown in Figure 3(c). Seven lipids, namely PC (36 : 1), TG (46 : 1), TG (56 : 6) PC (34 : 5), TG (56 : 8), TG (50 : 3), and DG (34 : 0), recorded VIP values exceeding 1.5 [65]. PC (36 : 1) was the most significant lipid in abalone tissues at different developmental stages, achieving a score close to 8.0, and it was also abundant in swimming veliger larvae. PC (34 : 5) was the most abundant in mature individuals, followed by juveniles and swimming veliger larvae, indicating that its content increases with the progression of abalone development.

**Figure 4:**
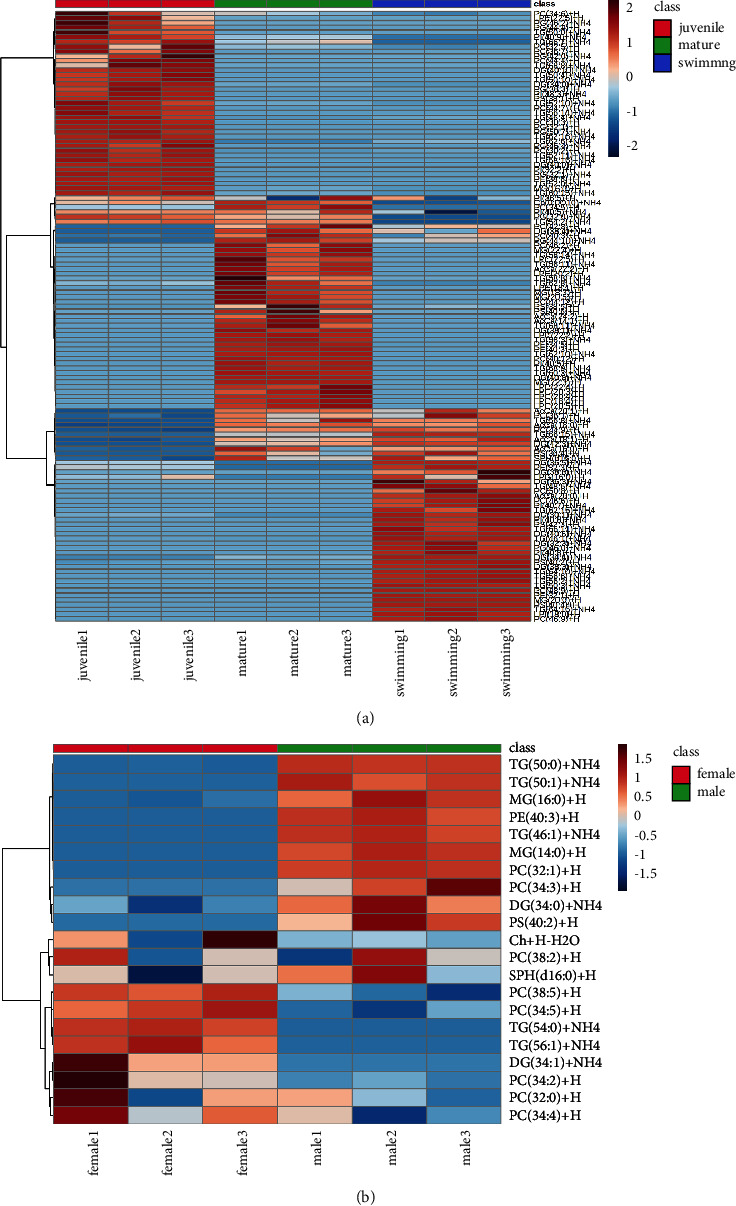
Heatmap showing the relative amounts of lipids in (a) abalone tissues at three different developmental stages (swimming veliger larvae, juveniles, and mature individuals) and (b) the hemolymph of female and male abalones.

Next, intrasample and intersample variations in hemolymph were visualized by PCA and PLS-DA, as shown in Figure S3(b) and Figure 3(b). On the PCA biplot, the two groups were well separated; PC1 explained 88.2% of the variance and PC2 explained 11.6% of the variance. Likewise, in the PLS-DA score plot, clear separation between female and male groups was observed, implying differences in lipid composition. Among the 21 lipids, the VIP score plot of the top 10 lipids based on the PLS-DA model is shown in Figure 3(d). The lipid species that contributed the most to the discrimination were DG (34 : 0) (VIP score > 3.0), followed by SPH (d16 : 0), and MG (16 : 0) (VIP score > 1.5). The heatmap in Figure 4 shows the tendencies of lipids identified in the tissue and hemolymph. Detailed data are reported in Figure S4.

### 3.5. Notable Lipid Synthesis Pathways

**Figure 5:**
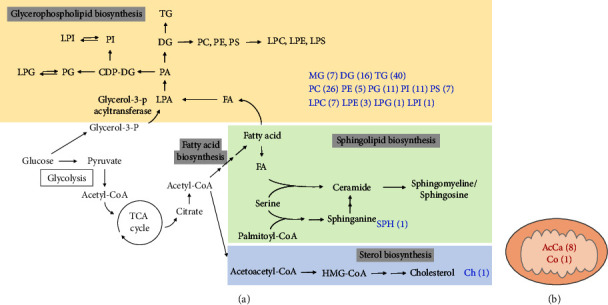
Schematic of the lipid biosynthetic pathways explored in the present study. (a) Lipid biosynthetic pathway. (b) Lipids are directly related to mitochondria. The blue and red texts are lipid classes detected in abalone tissue/hemolymph and the number of each lipid species.

A schematic of the notable lipid biosynthesis pathways explored in the present study is presented in Figure 5 [66, 67]. In the glycerophospholipid synthetic pathway, glycerolipids (MG, DG, and TG), phospholipids (PC, PE, PG, PI, and PS), and lysophospholipids (LPC, LPE, LPG, and LPI) were identified. The sphingolipid and sterol biosynthetic pathways are also part of the lipid synthesis pathway in abalone. In addition, acylcarnitine (AcCa) and coenzyme 10 are directly related to the mitochondria. AcCa is involved in fatty acid transport to the mitochondria, and Co participates in electron transfer [16].

The lipid biosynthetic pathways and species emphasized in the present study (Figure 5) will extend our understanding of *H. discus hannai* lipidome and can be applied in future studies. Based on these lipid biosynthetic pathways, the overall molecular mechanism of *H. discus hannai* development can be unveiled by assessing the expression of genes or proteins involved in lipid metabolism and synthesis (e.g., fatty acid synthase, carnitine palmitoyltransferase 1, acetyl-CoA carboxylase, and 3-hydroxy-3-methyl-glutaryl coenzyme A reductase) [68].

### 3.6. Significance of *H. discus hannai* Lipid Profiling

The present lipidomic study of *H. discus hannai* tissues and hemolymph allowed us to broaden the horizons of lipid profiling attempted thus far. In previous untargeted lipidomics studies of *H. discus hannai* tissues, Zhou et al. (2012) used Soxhlet, SC-CO_2_ extraction method, and identified 17 lipids using GC-MS. Lou et al. (2013) used the Folch extraction method and compared the composition of lipids such as cholesterol, triacylglycerol, free fatty acids, and phospholipids, and identified 32 fatty acids using GC-MS. The above two studies identified lipids using Wiley and NIST libraries. Zhang et al. (2018) used the Folch method and evaluated the effect of lipid composition on nutritional value using UPLC-ESI-Q-TOF-MS. In this study, 34 lipids were identified from 10 lipid classes, including phosphatidylcholine (PC), phosphatidylethanolamine (PE), phosphatidylserine (PS), phosphatidylglycerol (PG), phosphatidic acid (PA), lysophosphatidic acid (LPA), triacylglycerol (TG), steroids, terpenoids, and fatty acids. In the current study, we identified 132 positive lipids from 15 lipid classes through untargeted profiling, which is approximately four times greater than that recorded previously. In addition, through the scan-type lipidomics approach, 21 lipids from 8 lipid classes were identified in the hemolymph of *H. discus hannai*, which has not been previously studied. The details of the above are summarized in Table 1. Through this study, we were able to better understand the lipid composition of *H. discus hannai* than before. We believe this will provide insight into the properties of lipids involved in abalone growth.

## 4. Conclusions

Through an untargeted lipidomic approach using UHPLC-MS/MS, we identified 132 lipids in *H. discus hannai* tissues at different developmental stages. In addition, for the first time, we determined the lipid profile of the hemolymph of mature female and male *H. discus hannai*, identifying 21 lipids. Among the lipid species, TGs and PCs were the most abundant in both tissue (TG + PC = 42%) and hemolymph (TG + PC = 62%). In the tissue, TGs were the most abundant lipids (27%), whereas, in the hemolymph, PCs were the most abundant lipids (38%). In addition, the lipid composition of tissue differed depending on the developmental stage. Our results have several potential implications. First, lipid profiling can be helpful for screening lipidomic changes according to developmental stages; our untargeted approach revealed some potential lipid species with pivotal roles in abalone growth or development. Second, the present study, for the first time, identified lipids in the hemolymph of *H. discus hannai* and demonstrated that the lipid composition of hemolymph in abalone is similar to that in other living organisms. Overall, these findings will advance our understanding of the biology of this commercially important mollusk.

## Figures and Tables

**Table 1:** Comparison between the current study and previously published lipidomics studies of abalone (*Haliotis discus hannai*) tissue.

References	Extraction	Method	Software/libraries	Key findings (level identification)
Current study	(i) Neutral extraction CHCl_3_/MeOH	UHPLC-Q-Orbitrap-MS/MS	Lipidsearch	(i) 132 lipid species were identified
				(ii) The lipid composition of tissue at three different developmental stages included 15 lipid classes (TG, PC, etc)
	(ii) Acidic extraction CHCl_3_/MeOH/37% (1N) HCl			

Zhang et al., 2018 [33]	Folch method	UPLC-ESI-Q-TOF-MS	MS-DIAL and LIPID MAPS	(i) Thirty-four lipid species were identified (10 lipid classes)

Lou et al., 2013 [13]	Folch method	GC-MS	Wiley and NIST libraries	(i) The lipid composition of muscle and viscera tissues included total cholesterol, triacylglycerol, free fatty acids, and phospholipids
				(ii) Thirty-two fatty acids were identified

Zhou et al., 2012 [69]	(i) Soxhlet extraction	(i) TLC-FID (lipids)	NIST02 libraries	(i) The lipid composition of gonad tissue included TAG, phospholipids, cholesterol, and FAA
	(ii) SC-CO_2_ extraction	GC-MS (unsaponifiable/fatty acid methyl esters)		(ii) Composition of unsaponifiable lipids (4 sterol species) was examined
	(iii) Enzyme-assisted organic solvent method			(iii) Thirteen saponifiable fatty acids were identified
				(iv) Percent composition depends on the extraction method

## Data Availability

The data used to support the findings of this study are included within the article and the supplementary materials.

## Supplementary Materials

Supplementary Materials Table S1: List of identified lipids in tissue of abalone, *Haliotis discus hannai* at different developmental stages (swimming veliger larvae, juvenile, mature). Table S2: List of identified lipids in hemolymph of female and male abalone, *Haliotis discus hannai*. Table S3: List of the normalized area of lipids in tissue of abalone, *Haliotis discus hannai* at different developmental stages (swimming veliger larvae, juvenile, mature). Table S4: List of normalized area of lipids in hemolymph of female and male abalone, *Haliotis discus hannai.* Figure S1: Total ion current (TIC) chromatogram of the tissue samples: (a) Swimming veliger larvae, (b) Juvenile, (c) Mature. Figure S2: Total ion current (TIC) chromatogram of the hemolymph samples: (a) Female, (b) Male. Figure S3: PCA score plots of (a) tissues in three different developmental stages, (b) female and male hemolymph. Figure S4: The detailed heatmap of abalone tissue.
