# Supplementary material for: Lipid Profiling of Pacific Abalone (Haliotis discus hannai) at Different Developmental Stages Using Ultrahigh Performance Liquid Chromatography-Tandem Mass Spectrometry
Source: J Anal Methods Chem. 2022 Oct 17;2022:5822562. doi: 10.1155/2022/5822562 (PMC9592233; doi:10.1155/2022/5822562)

Supplementary materials

Lipid profiling of Pacific abalone (*Haliotis discus hannai*) at different developmental stages using ultra-high performance liquid chromatography-tandem mass spectrometry

HeyGene Lee^1,†^, MinJoong Joo^1,†^, Jong-Moon Park^2,†^, Mi Ae Kim^3,4^, JeongHun Mok^1^, Seong-Hyeon Cho^1^, Young Chang Sohn^3,^*, and Hookeun Lee^1,^*

^1^ College of Pharmacy, Gachon University, Incheon 21936, South Korea; iheygene@gmail.com(H.G.L); blackmash1@gachon.ac.kr (M.J.J); jeonghunmok@naver.com (J.H.M.); dk199@gachon.ac.kr (S.H.C.);

^2^ Basilbiotech, Incheon 22002, South Korea; bio4647@naver.com

^3^ Department of Marine Molecular Bioscience, Gangneung-Wonju National University, Gangneung 25457, South Korea; [kimmiaecho@gmail.com](mailto:kimmiaecho@gmail.com)

^4^  East Coast Life Sciences Institute, Gangneung-Wonju National University, Gangneung 25457, South Korea

*Correspondence: ycsohn@gwnu.ac.kr (Y.C.S); hklee@gachon.ac.kr (H.K.L);
Tel.: +82-33-640-2348 (Y.C.S.); +82-32-820-4927 (H.K.L.)

**Table of Content**

**Table S1 :** List of identified lipids in tissue of abalone, *Haliotis discus hannai* at different developmental stages (swimming veliger larvae, juvenile, mature)

**Table S2 :** List of identified lipids in hemolymph of female and male abalone, *Haliotis discus hannai*

**Table S3 :** List of the normalized area of lipids in tissue of abalone, *Haliotis discus hannai* at different developmental stages (swimming veliger larvae, juvenile, mature)

**Table S4 :** List of normalized area of lipids in hemolymph of female and male abalone, *Haliotis discus hannai*

**Figure S1 :** Total ion current (TIC) chromatogram of the tissue samples: (a) Swimming veliger larvae, (b) Juvenile, (c) Mature

**Figure S2 :** Total ion current (TIC) chromatogram of the hemolymph samples: (a) Female, (b) Male

**Figure S3 :** PCA score plots of (a) tissues in three different developmental stages, (b) female and male hemolymph

**Figure S4 :** The detailed heatmap of abalone tissue

**Table S1 :** List of identified lipids in tissue of abalone, *Haliotis discus hannai* at different developmental stages (swimming veliger larvae, juvenile, mature)

| **No.** | **Rt**  **(min)** | **Calculated**  **m/z** | **Theoretical**  **m/z** | **Error**  **(ppm)** | **Lipid name** | **Adduct**  **ion** | **swimming** | **juvenile** | **mature** |
| --- | --- | --- | --- | --- | --- | --- | --- | --- | --- |
|  |  |  |  |  |  |  | **Area_avg_ (%rsd)** | **Area_avg_ (%rsd)** | **Area_avg_ (%rsd)** |
| **1** | **2.56** | **370.2952** | **370.2952** | **0.00127** | **AcCa(14:1)+H** | **M+H** | **-** | **-** | **193902.2 (11.1)** |
| **2** | **3.42** | **400.3421** | **400.3421** | **0.00117** | **AcCa(16:0)+H** | **M+H** | **6833532.3 (8.2)** | **2409182.5 (4.9)** | **7062873.9 (39.6)** |
| **3** | **4.32** | **428.3734** | **428.3734** | **0.00124** | **AcCa(18:0)+H** | **M+H** | **2248489.3 (8.7)** | **601467.7 (11.0)** | **2353102.6 (5.8)** |
| **4** | **3.46** | **426.3578** | **426.3578** | **0.00110** | **AcCa(18:1)+H** | **M+H** | **4997163.8 (7.6)** | **1271795.8 (10.6)** | **3770820.0 (9.0)** |
| **5** | **5.67** | **456.4047** | **456.4047** | **0.00116** | **AcCa(20:0)+H** | **M+H** | **242720.5 (8.5)** | **-** | **-** |
| **6** | **4.36** | **454.3891** | **454.3891** | **0.00117** | **AcCa(20:1)+H** | **M+H** | **3346350.7 (29.0)** | **-** | **3597123.8 (0.8)** |
| **7** | **4.48** | **480.4047** | **480.4047** | **0.00110** | **AcCa(22:2)+H** | **M+H** | **-** | **-** | **1311032.4 (14.2)** |
| **8** | **5.75** | **508.4360** | **508.4360** | **0.00104** | **AcCa(24:2)+H** | **M+H** | **-** | **-** | **77121.0 (22.5)** |
| **9** | **21.64** | **880.7177** | **880.7177** | **0.00174** | **Co(0:0Q10)+NH4** | **M+NH4** | **2323121.5 (8.2)** | **8575345.5 (11.5)** | **12185869.8 (8.6)** |
| **10** | **14.09** | **556.4936** | **556.4936** | **0.00095** | **DG(30:1)+NH4** | **M+NH4** | **1288407.9 (4.8)** | **199422.0 (10.7)** | **-** |
| **11** | **12.20** | **580.4936** | **580.4936** | **0.00091** | **DG(32:3)+NH4** | **M+NH4** | **705850.3 (11.5)** | **-** | **-** |
| **12** | **17.93** | **614.5718** | **614.5718** | **0.00086** | **DG(34:0)+NH4** | **M+NH4** | **-** | **42141216.7 (8.7)** | **4247678.6 (1.2)** |
| **13** | **11.53** | **606.5092** | **606.5092** | **0.00087** | **DG(34:4)+NH4** | **M+NH4** | **414584.1 (0.9)** | **-** | **83920.1 (14.0)** |
| **14** | **13.73** | **632.5249** | **632.5249** | **0.00084** | **DG(36:5)+NH4** | **M+NH4** | **2397085.5 (1.5)** | **912883.2 (1.6)** | **-** |
| **15** | **17.09** | **662.5718** | **662.5718** | **0.00080** | **DG(38:4)+NH4** | **M+NH4** | **3067819.9 (49.6)** | **-** | **5420708.7 (14.5)** |
| **16** | **20.63** | **668.6188** | **668.6188** | **0.00079** | **DG(38:1)+NH4** | **M+NH4** | **-** | **-** | **594627.1 (6.6)** |
| **17** | **21.89** | **698.6657** | **698.6657** | **0.00076** | **DG(40:0)+NH4** | **M+NH4** | **-** | **385457.8 (1.7)** | **-** |
| **18** | **16.31** | **636.5562** | **636.5562** | **0.00083** | **DG(36:3)+NH4** | **M+NH4** | **588387.9 (41.9)** | **-** | **-** |
| **19** | **18.62** | **664.5875** | **664.5875** | **0.00080** | **DG(38:3)+NH4** | **M+NH4** | **3805126.3 (4.7)** | **-** | **-** |
| **20** | **15.52** | **686.5718** | **686.5718** | **0.00077** | **DG(40:6)+NH4** | **M+NH4** | **4662862.1 (4.2)** | **-** | **-** |
| **21** | **9.28** | **654.5092** | **654.5092** | **0.00081** | **DG(38:8)+NH4** | **M+NH4** | **491789.1 (42.3)** | **-** | **-** |
| **22** | **20.69** | **720.6501** | **720.6501** | **0.00074** | **DG(42:3)+NH4** | **M+NH4** | **641750.5 (2.2)** | **-** | **437667.3 (12.6)** |
| **23** | **12.08** | **682.5405** | **682.5405** | **0.00078** | **DG(40:8)+NH4** | **M+NH4** | **-** | **-** | **252837.6 (0.8)** |
| **24** | **8.79** | **678.5092** | **678.5092** | **0.00078** | **DG(40:10)+NH4** | **M+NH4** | **-** | **239506.0 (7.9)** | **-** |
| **25** | **2.41** | **516.3085** | **516.3085** | **0.00478** | **LPC(18:4)+H** | **M+H** | **-** | **-** | **318651.2 (7.4)** |
| **26** | **3.10** | **546.3554** | **546.3554** | **0.00452** | **LPC(20:3)+H** | **M+H** | **-** | **-** | **100541.0 (17.0)** |
| **27** | **2.77** | **544.3398** | **544.3398** | **0.00454** | **LPC(20:4)+H** | **M+H** | **-** | **-** | **3147393.9 (9.0)** |
| **28** | **2.51** | **542.3241** | **542.3241** | **0.00455** | **LPC(20:5)+H** | **M+H** | **-** | **-** | **2138204.3 (10.0)** |
| **29** | **4.70** | **576.4024** | **576.4024** | **0.00429** | **LPC(22:2)+H** | **M+H** | **-** | **-** | **813072.5 (2.9)** |
| **30** | **3.15** | **572.3711** | **572.3711** | **0.00432** | **LPC(22:4)+H** | **M+H** | **-** | **-** | **900841.8 (16.8)** |
| **31** | **2.83** | **570.3554** | **570.3554** | **0.00433** | **LPC(22:5)+H** | **M+H** | **-** | **-** | **2409354.9 (25.2)** |
| **32** | **3.43** | **480.3085** | **480.3085** | **0.00514** | **LPE(18:1)+H** | **M+H** | **-** | **-** | **514527.5 (11.9)** |
| **33** | **3.56** | **506.3241** | **506.3241** | **0.00488** | **LPE(20:2)+H** | **M+H** | **-** | **-** | **91752.6 (15.8)** |
| **34** | **2.79** | **528.3085** | **528.3085** | **0.00468** | **LPE(22:5)+H** | **M+H** | **-** | **751025.1 (44.8)** | **-** |
| **35** | **2.72** | **485.2874** | **485.2874** | **0.00715** | **LPG(16:0)+H** | **M+H** | **2452573.4 (46.4)** | **942180.2 (32.6)** | **-** |
| **36** | **3.62** | **601.3347** | **601.3347** | **0.00743** | **LPI(18:0)+H** | **M+H** | **1131491.9 (3.4)** | **-** | **-** |
| **37** | **4.03** | **331.2843** | **331.2843** | **0.00142** | **MG(16:0)+H** | **M+H** | **-** | **5856166.2 (2.3)** | **426056.4 (24.8)** |
| **38** | **2.93** | **353.2686** | **353.2686** | **0.00133** | **MG(18:3)+H** | **M+H** | **-** | **-** | **251564.5 (19.7)** |
| **39** | **6.92** | **387.3469** | **387.3469** | **0.00121** | **MG(20:0)+H** | **M+H** | **1120727.9 (0.1)** | **-** | **-** |
| **40** | **2.83** | **377.2686** | **377.2686** | **0.00125** | **MG(20:5)+H** | **M+H** | **-** | **-** | **174988.3 (16.6)** |
| **41** | **4.44** | **409.3312** | **409.3312** | **0.00115** | **MG(22:3)+H** | **M+H** | **-** | **-** | **107442.2 (0.2)** |
| **42** | **3.64** | **407.3156** | **407.3156** | **0.00115** | **MG(22:4)+H** | **M+H** | **-** | **-** | **105267.7 (16.1)** |
| **43** | **7.79** | **700.4912** | **700.4912** | **0.00353** | **PC(30:3)+H** | **M+H** | **69915.4 (7.4)** | **3015790.0 (3.5)** | **-** |
| **44** | **8.99** | **752.5225** | **752.5225** | **0.00328** | **PC(34:5)+H** | **M+H** | **3663397.1 (14.1)** | **67435475.0 (1.1)** | **210997231.8 (12.0)** |
| **45** | **18.55** | **840.6477** | **840.6477** | **0.00175** | **PC(40:3)+H** | **M+H** | **12480749.0 (45.4)** | **4854653.2 (14.5)** | **24681766.6 (18.0)** |
| **46** | **15.78** | **888.6477** | **888.6477** | **0.00165** | **PC(44:7)+H** | **M+H** | **-** | **968708.9 (6.5)** | **-** |
| **47** | **11.93** | **884.6164** | **884.6164** | **0.00166** | **PC(44:9)+H** | **M+H** | **2186192.1 (5.7)** | **502237.3 (14.9)** | **1984239.4 (5.0)** |
| **48** | **10.15** | **878.5694** | **878.5694** | **0.00281** | **PC(44:12)+H** | **M+H** | **-** | **-** | **171374.8 (18.0)** |
| **49** | **9.40** | **702.5068** | **702.5068** | **0.00352** | **PC(30:2)+H** | **M+H** | **587694.3 (11.3)** | **7760633.7 (8.8)** | **-** |
| **50** | **20.41** | **926.7572** | **926.7572** | **0.00159** | **PC(46:2)+H** | **M+H** | **-** | **-** | **139508.8 (21.5)** |
| **51** | **6.32** | **724.4912** | **724.4912** | **0.00341** | **PC(32:5)+H** | **M+H** | **66213.0 (9.3)** | **251068.8 (32.9)** | **105698.4 (5.2)** |
| **52** | **6.13** | **750.5068** | **750.5068** | **0.00329** | **PC(34:6)+H** | **M+H** | **53440.2 (18.7)** | **333699.9 (45.2)** | **134230.6 (24.4)** |
| **53** | **17.24** | **788.6164** | **788.6164** | **0.00186** | **PC(36:1)+H** | **M+H** | **317114136.1 (24.6)** | **140497761.5 (9.9)** | **275483437.8 (11.6)** |
| **54** | **6.88** | **776.5225** | **776.5225** | **0.00318** | **PC(36:7)+H** | **M+H** | **57020.1 (8.6)** | **2598153.3 (27.3)** | **-** |
| **55** | **6.26** | **822.5068** | **822.5068** | **0.00300** | **PC(40:12)+H** | **M+H** | **-** | **-** | **193844.0 (1.4)** |
| **56** | **11.18** | **858.6007** | **858.6007** | **0.00171** | **PC(42:8)+H** | **M+H** | **1466459.8 (16.2)** | **-** | **2630119.1 (33.1)** |
| **57** | **20.01** | **894.6946** | **894.6946** | **0.00164** | **PC(44:4)+H** | **M+H** | **-** | **249526.5 (32.9)** | **-** |
| **58** | **19.28** | **918.6946** | **918.6946** | **0.00160** | **PC(46:6)+H** | **M+H** | **216296.9 (13.2)** | **-** | **-** |
| **59** | **14.59** | **912.6477** | **912.6477** | **0.00161** | **PC(46:9)+H** | **M+H** | **102906.4 (3.5)** | **-** | **-** |
| **60** | **16.10** | **940.6790** | **940.6790** | **0.00156** | **PC(48:9)+H** | **M+H** | **95832.7 (0.6)** | **-** | **-** |
| **61** | **18.53** | **970.7259** | **970.7259** | **0.00151** | **PC(50:8)+H** | **M+H** | **314961.2 (26.4)** | **-** | **-** |
| **62** | **13.83** | **690.5068** | **690.5068** | **0.00358** | **PE(32:1)+H** | **M+H** | **1236670.8 (1.0)** | **-** | **-** |
| **63** | **9.54** | **686.4755** | **686.4755** | **0.00360** | **PE(32:3)+H** | **M+H** | **169789.3 (4.7)** | **71003.1 (5.8)** | **-** |
| **64** | **8.91** | **710.4755** | **710.4755** | **0.00348** | **PE(34:5)+H** | **M+H** | **1036192.5 (4.8)** | **1187995.3 (0.5)** | **14060179.1 (2.1)** |
| **65** | **11.38** | **764.5225** | **764.5225** | **0.00323** | **PE(38:6)+H** | **M+H** | **-** | **22281449.3 (2.7)** | **-** |
| **66** | **18.38** | **798.6007** | **798.6007** | **0.00184** | **PE(40:3)+H** | **M+H** | **-** | **-** | **5354348.1 (2.1)** |
| **67** | **9.28** | **740.5436** | **740.5436** | **0.00334** | **PG(32:0)+NH4** | **M+NH4** | **-** | **15973728.0 (6.3)** | **12037832.0 (12.8)** |
| **68** | **7.40** | **721.5014** | **721.5014** | **0.00481** | **PG(32:1)+H** | **M+H** | **-** | **85617.2 (0.6)** | **-** |
| **69** | **7.40** | **738.5280** | **738.5280** | **0.00334** | **PG(32:1)+NH4** | **M+NH4** | **-** | **129151.8 (2.3)** | **-** |
| **70** | **20.53** | **801.5640** | **801.5640** | **0.00308** | **PG(38:3)+H** | **M+H** | **-** | **208256.2 (13.1)** | **-** |
| **71** | **8.61** | **888.5749** | **888.5749** | **0.00278** | **PG(44:10)+NH4** | **M+NH4** | **27371.3 (10.6)** | **-** | **55550.1 (7.4)** |
| **72** | **17.38** | **880.7001** | **880.7001** | **0.00280** | **PG(42:0)+NH4** | **M+NH4** | **-** | **150811.2 (46.5)** | **-** |
| **73** | **21.68** | **883.6423** | **883.6423** | **0.00280** | **PG(44:4)+H** | **M+H** | **-** | **231549.1 (21.9)** | **-** |
| **74** | **19.79** | **936.7627** | **936.7627** | **0.00157** | **PG(46:0)+NH4** | **M+NH4** | **185179.8 (7.8)** | **-** | **-** |
| **75** | **18.94** | **932.7314** | **932.7314** | **0.00265** | **PG(46:2)+NH4** | **M+NH4** | **-** | **550909.5 (43.4)** | **-** |
| **76** | **15.97** | **928.7001** | **928.7001** | **0.00266** | **PG(46:4)+NH4** | **M+NH4** | **-** | **129961.0 (12.8)** | **-** |
| **77** | **15.44** | **978.7158** | **978.7158** | **0.00252** | **PG(50:7)+NH4** | **M+NH4** | **-** | **233991.6 (5.3)** | **-** |
| **78** | **9.98** | **807.5018** | **807.5018** | **0.00554** | **PI(32:2)+H** | **M+H** | **-** | **958617.1 (2.1)** | **-** |
| **79** | **13.60** | **913.5801** | **913.5801** | **0.00380** | **PI(40:5)+H** | **M+H** | **-** | **-** | **119526.8 (3.0)** |
| **80** | **11.79** | **885.5488** | **885.5488** | **0.00392** | **PI(38:5)+H** | **M+H** | **-** | **-** | **-** |
| **81** | **7.28** | **898.5440** | **898.5440** | **0.00386** | **PI(38:7)+NH4** | **M+NH4** | **-** | **-** | **-** |
| **82** | **15.35** | **906.6066** | **906.6066** | **0.00383** | **PI(38:3)+NH4** | **M+NH4** | **-** | **235636.3 (7.5)** | **-** |
| **83** | **9.49** | **926.5753** | **926.5753** | **0.00374** | **PI(40:7)+NH4** | **M+NH4** | **257992.9 (14.8)** | **-** | **-** |
| **84** | **7.73** | **907.5331** | **907.5331** | **0.00382** | **PI(40:8)+H** | **M+H** | **29063.5 (7.1)** | **-** | **-** |
| **85** | **7.73** | **924.5597** | **924.5597** | **0.00375** | **PI(40:8)+NH4** | **M+NH4** | **225922.4 (6.6)** | **-** | **-** |
| **86** | **6.44** | **922.5440** | **922.5440** | **0.00376** | **PI(40:9)+NH4** | **M+NH4** | **-** | **71360.5 (12.2)** | **26149.5 (7.6)** |
| **87** | **9.26** | **857.5175** | **857.5175** | **0.00521** | **PI(36:5)+H** | **M+H** | **314640.0 (13.3)** | **359973.0 (3.9)** | **331269.1 (22.0)** |
| **88** | **11.81** | **930.6066** | **930.6066** | **0.00373** | **PI(40:5)+NH4** | **M+NH4** | **492252.9 (45.3)** | **898321.5 (1.4)** | **935051.7 (14.5)** |
| **89** | **11.73** | **810.5280** | **810.5280** | **0.00305** | **PS(38:5)+H** | **M+H** | **926759.2 (27.7)** | **-** | **6040323.4 (48.5)** |
| **90** | **19.00** | **818.5906** | **818.5906** | **0.00302** | **PS(38:1)+H** | **M+H** | **-** | **657115.8 (5.9)** | **-** |
| **91** | **18.97** | **844.6062** | **844.6062** | **0.00292** | **PS(40:2)+H** | **M+H** | **1703957.1 (2.0)** | **448805.6 (8.9)** | **497551.0 (3.8)** |
| **92** | **15.87** | **840.5749** | **840.5749** | **0.00294** | **PS(40:4)+H** | **M+H** | **661633.1 (0.5)** | **-** | **-** |
| **93** | **19.00** | **870.6219** | **870.6219** | **0.00284** | **PS(42:3)+H** | **M+H** | **211984.4 (8.8)** | **-** | **-** |
| **94** | **11.79** | **784.5123** | **784.5123** | **0.00315** | **PS(36:4)+H** | **M+H** | **1037000.3 (15.8)** | **-** | **640326.8 (43.1)** |
| **95** | **12.68** | **836.5436** | **836.5436** | **0.00295** | **PS(40:6)+H** | **M+H** | **-** | **-** | **262116.8 (40.3)** |
| **96** | **2.34** | **274.2741** | **274.2741** | **0.00193** | **SPH(d16:0)+H** | **M+H** | **74290504.3 (9.1)** | **46087127.4 (3.7)** | **68115299.0 (15.4)** |
| **97** | **20.60** | **788.6763** | **788.6763** | **0.00067** | **TG(46:4)+NH4** | **M+NH4** | **1863628.9 (8.5)** | **22038226.1 (10.0)** | **-** |
| **98** | **20.24** | **812.6763** | **812.6763** | **0.00065** | **TG(48:6)+NH4** | **M+NH4** | **2781601.1 (23.1)** | **-** | **-** |
| **99** | **22.29** | **794.7232** | **794.7232** | **0.00067** | **TG(46:1)+NH4** | **M+NH4** | **114621807.0 (3.7)** | **4282176.6 (4.9)** | **-** |
| **100** | **18.82** | **860.6763** | **860.6763** | **0.00062** | **TG(52:10)+NH4** | **M+NH4** | **-** | **5516694.9 (14.5)** | **512421.2 (8.0)** |
| **101** | **23.61** | **852.8015** | **852.8015** | **0.00179** | **TG(50:0)+NH4** | **M+NH4** | **-** | **2017946.5 (30.2)** | **-** |
| **102** | **23.88** | **880.8328** | **880.8328** | **0.00174** | **TG(52:0)+NH4** | **M+NH4** | **-** | **1397369.2 (1.9)** | **-** |
| **103** | **23.30** | **904.8328** | **904.8328** | **0.00169** | **TG(54:2)+NH4** | **M+NH4** | **-** | **11639254.5 (6.0)** | **11118875.3 (2.1)** |
| **104** | **23.86** | **958.8797** | **958.8797** | **0.00160** | **TG(58:3)+NH4** | **M+NH4** | **-** | **-** | **8766188.0 (4.2)** |
| **105** | **23.18** | **1082.9110** | **1082.9110** | **0.00141** | **TG(68:11)+NH4** | **M+NH4** | **-** | **-** | **229078.2 (22.1)** |
| **106** | **22.30** | **846.7545** | **846.7545** | **0.00063** | **TG(50:3)+NH4** | **M+NH4** | **68556873.8 (1.7)** | **-** | **-** |
| **107** | **21.81** | **844.7389** | **844.7389** | **0.00063** | **TG(50:4)+NH4** | **M+NH4** | **-** | **19343048.4 (11.6)** | **-** |
| **108** | **22.48** | **924.8015** | **924.8015** | **0.00165** | **TG(56:6)+NH4** | **M+NH4** | **112734791.4 (3.0)** | **-** | **-** |
| **109** | **24.05** | **1062.9423** | **1062.9423** | **0.00144** | **TG(66:7)+NH4** | **M+NH4** | **-** | **306330.5 (9.3)** | **146192.6 (15.4)** |
| **110** | **17.57** | **832.6450** | **832.6450** | **0.00064** | **TG(50:10)+NH4** | **M+NH4** | **-** | **650986.9 (11.4)** | **-** |
| **111** | **17.81** | **858.6606** | **858.6606** | **0.00062** | **TG(52:11)+NH4** | **M+NH4** | **-** | **5760923.8 (5.1)** | **-** |
| **112** | **23.81** | **932.8641** | **932.8641** | **0.00164** | **TG(56:2)+NH4** | **M+NH4** | **7056913.1 (3.7)** | **-** | **-** |
| **113** | **24.19** | **986.9110** | **986.9110** | **0.00155** | **TG(60:3)+NH4** | **M+NH4** | **-** | **-** | **991026.0 (2.8)** |
| **114** | **22.06** | **948.8015** | **948.8015** | **0.00161** | **TG(58:8)+NH4** | **M+NH4** | **-** | **-** | **99251594.0 (49.8)** |
| **115** | **21.42** | **920.7702** | **920.7702** | **0.00058** | **TG(56:8)+NH4** | **M+NH4** | **66169580.8 (0.6)** | **-** | **70008903.9 (32.8)** |
| **116** | **23.26** | **1006.8797** | **1006.8797** | **0.00152** | **TG(62:7)+NH4** | **M+NH4** | **-** | **-** | **-** |
| **117** | **23.76** | **1008.8954** | **1008.8954** | **0.00152** | **TG(62:6)+NH4** | **M+NH4** | **659238.1 (12.3)** | **2722628.7 (4.1)** | **-** |
| **118** | **22.95** | **1004.8641** | **1004.8641** | **0.00152** | **TG(62:8)+NH4** | **M+NH4** | **-** | **2317866.5 (14.3)** | **9278340.6 (27.0)** |
| **119** | **22.14** | **1000.8328** | **1000.8328** | **0.00153** | **TG(62:10)+NH4** | **M+NH4** | **-** | **-** | **17193873.0 (1.5)** |
| **120** | **22.59** | **1028.8641** | **1028.8641** | **0.00149** | **TG(64:10)+NH4** | **M+NH4** | **1060303.9 (2.2)** | **-** | **-** |
| **121** | **19.90** | **914.7232** | **914.7232** | **0.00058** | **TG(56:11)+NH4** | **M+NH4** | **-** | **-** | **7517454.6 (20.1)** |
| **122** | **18.55** | **936.7076** | **936.7076** | **0.00057** | **TG(58:14)+NH4** | **M+NH4** | **-** | **-** | **1901030.6 (10.6)** |
| **123** | **18.88** | **962.7232** | **962.7232** | **0.00055** | **TG(60:15)+NH4** | **M+NH4** | **76371.2 (16.2)** | **19262957.5 (5.5)** | **1995497.4 (11.9)** |
| **124** | **19.71** | **990.7545** | **990.7545** | **0.00053** | **TG(62:15)+NH4** | **M+NH4** | **378217.5 (16.9)** | **-** | **-** |
| **125** | **19.29** | **988.7389** | **988.7389** | **0.00054** | **TG(62:16)+NH4** | **M+NH4** | **-** | **4087668.7 (3.5)** | **-** |
| **126** | **20.25** | **1018.7858** | **1018.7858** | **0.00052** | **TG(64:15)+NH4** | **M+NH4** | **801565.2 (4.1)** | **-** | **-** |
| **127** | **23.60** | **1086.9423** | **1086.9423** | **0.00141** | **TG(68:9)+NH4** | **M+NH4** | **-** | **-** | **159015.7 (2.3)** |
| **128** | **22.94** | **1054.8797** | **1054.8797** | **0.00145** | **TG(66:11)+NH4** | **M+NH4** | **285084.3 (9.4)** | **-** | **-** |
| **129** | **20.80** | **1046.8171** | **1046.8171** | **0.00146** | **TG(66:15)+NH4** | **M+NH4** | **497375.3 (1.7)** | **-** | **241199.4 (2.1)** |
| **130** | **23.74** | **1088.9580** | **1088.9580** | **0.00141** | **TG(68:8)+NH4** | **M+NH4** | **-** | **118566.7 (48.9)** | **-** |
| **131** | **17.22** | **908.6763** | **908.6763** | **0.00058** | **TG(56:14)+NH4** | **M+NH4** | **-** | **2364532.9 (10.6)** | **-** |
| **132** | **17.78** | **934.6919** | **934.6919** | **0.00057** | **TG(58:15)+NH4** | **M+NH4** | **-** | **402270.7 (5.8)** | **-** |

*Lipids that have Area %rsd above 50 were removed.

*“-‘ means ‘undetected at least 2 of triplicate’.

*Nomenclature : “AcCa, acyl cartinine”, “Co, coenzyme”, “DG, diglyceride”, “LPC, lysophosphatidylcholine”, “LPE, lysophosphatidylethanolamine”, “LPG, lysophosphatidylglycerol”, “LPI, lysophosphatidylinositol”, “MG, monoglyceride”, “PC, phosphatidylcholine”, “PE, phosphatidylethanolamine”, “PG, phosphatidylglycerol”, “PI, phosphatidylinositol”, “PS, phosphatidylserine”, “SPH, sphinganine ”, “TG, triglyceride”

| **No.** | **Rt**  **(min)** | **Calculated**  **m/z** | **Theoretical**  **m/z** | **Error**  **(ppm)** | **Lipid name** | **Adduct**  **ion** | **female** | **male** |
| --- | --- | --- | --- | --- | --- | --- | --- | --- |
|  |  |  |  |  |  |  | **Area_avg_ (%rsd)** | **Area_avg_ (%rsd)** |
| **1** | **13.28** | **369.3516** | **369.3516** | **0.00414** | **Ch+H-H2O** | **M+H-H2O** | **480809.8 (47.2)** | **363810.5 (5.5)** |
| **2** | **18.34** | **614.5718** | **614.5718** | **0.00086** | **DG(34:0)+NH4** | **M+NH4** | **29397356.6 (29.9)** | **63308861.6 (16.7)** |
| **3** | **18.52** | **612.5562** | **612.5562** | **0.00087** | **DG(34:1)+NH4** | **M+NH4** | **469151.9 (45.6)** | **-** |
| **4** | **3.41** | **303.2530** | **303.2530** | **0.00155** | **MG(14:0)+H** | **M+H** | **-** | **478705.2 (6.3)** |
| **5** | **4.28** | **331.2843** | **331.2843** | **0.00142** | **MG(16:0)+H** | **M+H** | **4121854.8 (27.0)** | **29191708.3 (14.4)** |
| **6** | **17.80** | **734.5694** | **734.5694** | **0.00336** | **PC(32:0)+H** | **M+H** | **706152.5 (42.7)** | **574821.1 (22.4)** |
| **7** | **15.18** | **732.5538** | **732.5538** | **0.00337** | **PC(32:1)+H** | **M+H** | **-** | **1835777.8 (3.8)** |
| **8** | **15.39** | **758.5694** | **758.5694** | **0.00326** | **PC(34:2)+H** | **M+H** | **784139.3 (30.9)** | **485620.8 (7.3)** |
| **9** | **13.28** | **756.5538** | **756.5538** | **0.00326** | **PC(34:3)+H** | **M+H** | **-** | **82006.6 (43.0)** |
| **10** | **11.98** | **754.5381** | **754.5381** | **0.00327** | **PC(34:4)+H** | **M+H** | **667508.7 (8.5)** | **571302.8 (9.5)** |
| **11** | **9.86** | **752.5225** | **752.5225** | **0.00328** | **PC(34:5)+H** | **M+H** | **132847.8 (2.8)** | **108810.2 (4.9)** |
| **12** | **19.48** | **814.6320** | **814.6320** | **0.00180** | **PC(38:2)+H** | **M+H** | **421948.4 (28.2)** | **418101.0 (35.1)** |
| **13** | **13.99** | **808.5851** | **808.5851** | **0.00182** | **PC(38:5)+H** | **M+H** | **2956509.4 (3.8)** | **1834790.4 (17.8)** |
| **14** | **18.93** | **798.6007** | **798.6007** | **0.00184** | **PE(40:3)+H** | **M+H** | **-** | **55668.6 (7.2)** |
| **15** | **19.33** | **844.6062** | **844.6062** | **0.00292** | **PS(40:2)+H** | **M+H** | **-** | **34833.9 (33.3)** |
| **16** | **2.41** | **274.2741** | **274.2741** | **0.00193** | **SPH(d16:0)+H** | **M+H** | **180144971.5 (13.5)** | **206885218.2 (9.9)** |
| **17** | **22.57** | **794.7232** | **794.7232** | **0.00067** | **TG(46:1)+NH4** | **M+NH4** | **-** | **133421.0 (4.8)** |
| **18** | **23.68** | **852.8015** | **852.8015** | **0.00179** | **TG(50:0)+NH4** | **M+NH4** | **527812.1 (11.9)** | **8312974.4 (1.8)** |
| **19** | **23.40** | **850.7858** | **850.7858** | **0.00062** | **TG(50:1)+NH4** | **M+NH4** | **-** | **334708.2 (8.0)** |
| **20** | **24.37** | **908.8641** | **908.8641** | **0.00168** | **TG(54:0)+NH4** | **M+NH4** | **567887.3 (5.2)** | **-** |
| **21** | **24.33** | **934.8797** | **934.8797** | **0.00164** | **TG(56:1)+NH4** | **M+NH4** | **103418.7 (13.6)** | **-** |

**Table S2 :** List of identified lipids in hemolymph of female and male abalone, *Haliotis discus hannai*

*Lipids that have Area %rsd above 50 were removed.

*“-‘ Undetected at least 2 of triplicate.

*Nomenclature : “Ch, cholesterol”, “DG, diglyceride”, “MG, monoglyceride”, “PC, phosphatidylcholine”, “PE, phosphatidylethanolamine”, “PS, phosphatidylserine”, “SPH, sphinganine ”, “TG, triglyceride”

**Table S3 :** List of the normalized area of lipids in tissue of abalone, *Haliotis discus hannai* at different developmental stages (swimming veliger larvae, juvenile, mature)

| **No.** | **Lipid name** | **swimming** | | | **juvenile** | | | **mature** | | |
| --- | --- | --- | --- | --- | --- | --- | --- | --- | --- | --- |
|  |  | **swimming1** | **swimming2** | **swimming3** | **juvenile1** | **juvenile2** | **juvenile3** | **mature1** | **mature2** | **mature3** |
| **1** | **AcCa(14:1)+H** | **35811.22** | **35811.22** | **35811.22** | **35811.22** | **35811.22** | **35811.22** | **183945.7** | **218704.8** | **179056.1** |
| **2** | **AcCa(16:0)+H** | **6425428** | **6602513** | **7472657** | **2519118** | **2284871** | **2423559** | **9230985** | **8056005** | **3901632** |
| **3** | **AcCa(18:0)+H** | **2140159** | **2130022** | **2475286** | **535468.9** | **601467.7** | **667466.4** | **2353103** | **2215728** | **2490477** |
| **4** | **AcCa(18:1)+H** | **4919623** | **4663252** | **5408616** | **1271796** | **1137345** | **1406247** | **3977236** | **3379512** | **3955712** |
| **5** | **AcCa(20:0)+H** | **222170.5** | **242720.5** | **263270.5** | **44434.11** | **44434.11** | **44434.11** | **44434.11** | **44434.11** | **44434.11** |
| **6** | **AcCa(20:1)+H** | **2376349** | **4316353** | **3346351** | **475269.8** | **475269.8** | **475269.8** | **3582906** | **3577276** | **3631190** |
| **7** | **AcCa(22:2)+H** | **225051.3** | **225051.3** | **225051.3** | **225051.3** | **225051.3** | **225051.3** | **1496808** | **1125257** | **1311032** |
| **8** | **AcCa(24:2)+H** | **11813.65** | **11813.65** | **11813.65** | **11813.65** | **11813.65** | **11813.65** | **59068.23** | **93595.88** | **78698.84** |
| **9** | **Co(0:0Q10)+NH4** | **2157593** | **2280111** | **2531660** | **8298791** | **9669167** | **7758078** | **13200000** | **12300000** | **11100000** |
| **10** | **DG(30:1)+NH4** | **1331856** | **1218131** | **1315237** | **178169.8** | **220674.1** | **199422** | **35633.96** | **35633.96** | **35633.96** |
| **11** | **DG(32:3)+NH4** | **705850.3** | **787304.3** | **624396.3** | **124879.3** | **124879.3** | **124879.3** | **124879.3** | **124879.3** | **124879.3** |
| **12** | **DG(34:0)+NH4** | **839494** | **839494** | **839494** | **38100000** | **45300000** | **43100000** | **4247679** | **4197470** | **4297887** |
| **13** | **DG(34:4)+NH4** | **418306.1** | **410862.1** | **414584.1** | **14440.1** | **14440.1** | **14440.1** | **95639.71** | **72200.52** | **83920.12** |
| **14** | **DG(36:5)+NH4** | **2403286** | **2429022** | **2358948** | **897981.6** | **912883.2** | **927784.7** | **179596.3** | **179596.3** | **179596.3** |
| **15** | **DG(38:4)+NH4** | **3067820** | **1546634** | **4589006** | **309326.8** | **309326.8** | **309326.8** | **5420709** | **6207959** | **4633458** |
| **16** | **DG(38:1)+NH4** | **111020.5** | **111020.5** | **111020.5** | **111020.5** | **111020.5** | **111020.5** | **634151.7** | **555102.4** | **594627.1** |
| **17** | **DG(40:0)+NH4** | **75795.46** | **75795.46** | **75795.46** | **378977.3** | **385457.8** | **391938.3** | **75795.46** | **75795.46** | **75795.46** |
| **18** | **DG(36:3)+NH4** | **832502.9** | **593611.5** | **339049.3** | **67809.85** | **67809.85** | **67809.85** | **67809.85** | **67809.85** | **67809.85** |
| **19** | **DG(38:3)+NH4** | **3672007** | **3733756** | **4009617** | **734401.4** | **734401.4** | **734401.4** | **734401.4** | **734401.4** | **734401.4** |
| **20** | **DG(40:6)+NH4** | **4860352** | **4465372** | **4662862** | **893074.5** | **893074.5** | **893074.5** | **893074.5** | **893074.5** | **893074.5** |
| **21** | **DG(38:8)+NH4** | **345029.1** | **400616.4** | **729721.6** | **69005.83** | **69005.83** | **69005.83** | **69005.83** | **69005.83** | **69005.83** |
| **22** | **DG(42:3)+NH4** | **656792** | **639867.1** | **628592.5** | **78987.52** | **78987.52** | **78987.52** | **394937.6** | **418141.1** | **499923.2** |
| **23** | **DG(40:8)+NH4** | **50154.66** | **50154.66** | **50154.66** | **50154.66** | **50154.66** | **50154.66** | **252837.6** | **254901.9** | **250773.3** |
| **24** | **DG(40:10)+NH4** | **44105.95** | **44105.95** | **44105.95** | **220529.8** | **239506** | **258482.2** | **44105.95** | **44105.95** | **44105.95** |
| **25** | **LPC(18:4)+H** | **58999.23** | **58999.23** | **58999.23** | **58999.23** | **58999.23** | **58999.23** | **294996.2** | **318651.2** | **342306.2** |
| **26** | **LPC(20:3)+H** | **16683.83** | **16683.83** | **16683.83** | **16683.83** | **16683.83** | **16683.83** | **83419.15** | **100541** | **117662.8** |
| **27** | **LPC(20:4)+H** | **594869.9** | **594869.9** | **594869.9** | **594869.9** | **594869.9** | **594869.9** | **2974349** | **2992782** | **3475051** |
| **28** | **LPC(20:5)+H** | **385056.5** | **385056.5** | **385056.5** | **385056.5** | **385056.5** | **385056.5** | **1925282** | **2138204** | **2351126** |
| **29** | **LPC(22:2)+H** | **157854.1** | **157854.1** | **157854.1** | **157854.1** | **157854.1** | **157854.1** | **813072.5** | **836874.6** | **789270.4** |
| **30** | **LPC(22:4)+H** | **157861.3** | **157861.3** | **157861.3** | **157861.3** | **157861.3** | **157861.3** | **840241.3** | **789306.5** | **1072978** |
| **31** | **LPC(22:5)+H** | **392658.2** | **392658.2** | **392658.2** | **392658.2** | **392658.2** | **392658.2** | **3100274** | **1963291** | **2164500** |
| **32** | **LPE(18:1)+H** | **92355.99** | **92355.99** | **92355.99** | **92355.99** | **92355.99** | **92355.99** | **581750.6** | **500051.8** | **461780** |
| **33** | **LPE(20:2)+H** | **15785.85** | **15785.85** | **15785.85** | **15785.85** | **15785.85** | **15785.85** | **107527.9** | **78929.25** | **88800.58** |
| **34** | **LPE(22:5)+H** | **73788.25** | **73788.25** | **73788.25** | **1003685** | **880449.2** | **368941.3** | **73788.25** | **73788.25** | **73788.25** |
| **35** | **LPG(16:0)+H** | **2515182** | **1285468** | **3557070** | **778562** | **751698.1** | **1296281** | **150339.6** | **150339.6** | **150339.6** |
| **36** | **LPI(18:0)+H** | **1131492** | **1169872** | **1093112** | **218622.3** | **218622.3** | **218622.3** | **218622.3** | **218622.3** | **218622.3** |
| **37** | **MG(16:0)+H** | **60940.03** | **60940.03** | **60940.03** | **5992103** | **5856166** | **5720229** | **304700.1** | **496215.8** | **477253.3** |
| **38** | **MG(18:3)+H** | **41626.22** | **41626.22** | **41626.22** | **41626.22** | **41626.22** | **41626.22** | **305520.1** | **241042.3** | **208131.1** |
| **39** | **MG(20:0)+H** | **1120728** | **1120106** | **1121350** | **224021.2** | **224021.2** | **224021.2** | **224021.2** | **224021.2** | **224021.2** |
| **40** | **MG(20:5)+H** | **29037.15** | **29037.15** | **29037.15** | **29037.15** | **29037.15** | **29037.15** | **203181.2** | **176598** | **145185.8** |
| **41** | **MG(22:3)+H** | **21450.31** | **21450.31** | **21450.31** | **21450.31** | **21450.31** | **21450.31** | **107632.8** | **107442.2** | **107251.6** |
| **42** | **MG(22:4)+H** | **17182.46** | **17182.46** | **17182.46** | **17182.46** | **17182.46** | **17182.46** | **112782** | **85912.31** | **117108.8** |
| **43** | **PC(30:3)+H** | **64748.13** | **69915.43** | **75082.72** | **3069671** | **3083725** | **2893974** | **12949.63** | **12949.63** | **12949.63** |
| **44** | **PC(34:5)+H** | **4179711** | **3147084** | **3663397** | **68200000** | **67400000** | **66700000** | **2.11E+08** | **1.86E+08** | **2.36E+08** |
| **45** | **PC(40:3)+H** | **6142585** | **14200000** | **17100000** | **4854653** | **5558459** | **4150847** | **20200000** | **29100000** | **24700000** |
| **46** | **PC(44:7)+H** | **181227.1** | **181227.1** | **181227.1** | **1031282** | **968708.9** | **906135.6** | **181227.1** | **181227.1** | **181227.1** |
| **47** | **PC(44:9)+H** | **2310831** | **2061553** | **2186192** | **577020.3** | **502237.3** | **427454.3** | **1885282** | **1984239** | **2083196** |
| **48** | **PC(44:12)+H** | **28050.21** | **28050.21** | **28050.21** | **28050.21** | **28050.21** | **28050.21** | **202092.4** | **171780.9** | **140251.1** |
| **49** | **PC(30:2)+H** | **521266.2** | **587694.3** | **654122.5** | **8308646** | **6990189** | **7983067** | **104253.2** | **104253.2** | **104253.2** |
| **50** | **PC(46:2)+H** | **20979.33** | **20979.33** | **20979.33** | **20979.33** | **20979.33** | **20979.33** | **158679.5** | **104896.7** | **154950.2** |
| **51** | **PC(32:5)+H** | **66213** | **60087.65** | **72338.35** | **280236.7** | **157952.9** | **315016.8** | **105698.4** | **111185.3** | **100211.5** |
| **52** | **PC(34:6)+H** | **53440.16** | **43421.4** | **63458.92** | **484597.1** | **333700** | **182802.8** | **167014.5** | **134230.6** | **101446.8** |
| **53** | **PC(36:1)+H** | **2.33E+08** | **3.86E+08** | **3.32E+08** | **1.40E+08** | **1.55E+08** | **1.27E+08** | **3.07E+08** | **2.43E+08** | **2.75E+08** |
| **54** | **PC(36:7)+H** | **51659.2** | **61328.94** | **58072.12** | **2966139** | **1780570** | **3047751** | **10331.84** | **10331.84** | **10331.84** |
| **55** | **PC(40:12)+H** | **38233.66** | **38233.66** | **38233.66** | **38233.66** | **38233.66** | **38233.66** | **196756.5** | **191168.3** | **193607.1** |
| **56** | **PC(42:8)+H** | **1228909** | **1704011** | **1466460** | **245781.8** | **245781.8** | **245781.8** | **1758331** | **2630119** | **3501907** |
| **57** | **PC(44:4)+H** | **30935.74** | **30935.74** | **30935.74** | **154678.7** | **298652.5** | **295248.2** | **30935.74** | **30935.74** | **30935.74** |
| **58** | **PC(46:6)+H** | **187766.7** | **216296.9** | **244827.1** | **37553.33** | **37553.33** | **37553.33** | **37553.33** | **37553.33** | **37553.33** |
| **59** | **PC(46:9)+H** | **100909.3** | **107109.2** | **100700.7** | **20140.14** | **20140.14** | **20140.14** | **20140.14** | **20140.14** | **20140.14** |
| **60** | **PC(48:9)+H** | **95280.51** | **96384.94** | **95832.72** | **19056.1** | **19056.1** | **19056.1** | **19056.1** | **19056.1** | **19056.1** |
| **61** | **PC(50:8)+H** | **231732.8** | **398189.6** | **314961.2** | **46346.56** | **46346.56** | **46346.56** | **46346.56** | **46346.56** | **46346.56** |
| **62** | **PE(32:1)+H** | **1248585** | **1224757** | **1236671** | **244951.4** | **244951.4** | **244951.4** | **244951.4** | **244951.4** | **244951.4** |
| **63** | **PE(32:3)+H** | **161853.3** | **177725.3** | **169789.3** | **75149.86** | **71003.1** | **66856.35** | **13371.27** | **13371.27** | **13371.27** |
| **64** | **PE(34:5)+H** | **979333.7** | **1066653** | **1062591** | **1192671** | **1181250** | **1190065** | **13800000** | **14300000** | **14100000** |
| **65** | **PE(38:6)+H** | **4340000** | **4340000** | **4340000** | **22300000** | **21700000** | **22900000** | **4340000** | **4340000** | **4340000** |
| **66** | **PE(40:3)+H** | **1048549** | **1048549** | **1048549** | **1048549** | **1048549** | **1048549** | **5242743** | **5354348** | **5465953** |
| **67** | **PG(32:0)+NH4** | **2100000** | **2100000** | **2100000** | **16800000** | **16300000** | **14900000** | **12100000** | **10500000** | **13500000** |
| **68** | **PG(32:1)+H** | **17016.04** | **17016.04** | **17016.04** | **85617.17** | **86154.13** | **85080.21** | **17016.04** | **17016.04** | **17016.04** |
| **69** | **PG(32:1)+NH4** | **25228.59** | **25228.59** | **25228.59** | **132160.6** | **126142.9** | **129151.8** | **25228.59** | **25228.59** | **25228.59** |
| **70** | **PG(38:3)+H** | **36205.67** | **36205.67** | **36205.67** | **181028.4** | **235484.1** | **208256.2** | **36205.67** | **36205.67** | **36205.67** |
| **71** | **PG(44:10)+NH4** | **24033.03** | **29131.14** | **28949.87** | **4806.606** | **4806.606** | **4806.606** | **56697.39** | **58970.09** | **50982.92** |
| **72** | **PG(42:0)+NH4** | **16142.08** | **16142.08** | **16142.08** | **80710.38** | **150811.2** | **220912** | **16142.08** | **16142.08** | **16142.08** |
| **73** | **PG(44:4)+H** | **36187.29** | **36187.29** | **36187.29** | **282161.9** | **231549.2** | **180936.4** | **36187.29** | **36187.29** | **36187.29** |
| **74** | **PG(46:0)+NH4** | **185179.8** | **199592.5** | **170767.2** | **34153.43** | **34153.43** | **34153.43** | **34153.43** | **34153.43** | **34153.43** |
| **75** | **PG(46:2)+NH4** | **62359.04** | **62359.04** | **62359.04** | **790023.8** | **550909.5** | **311795.2** | **62359.04** | **62359.04** | **62359.04** |
| **76** | **PG(46:4)+NH4** | **22677.95** | **22677.95** | **22677.95** | **129961** | **113389.8** | **146532.2** | **22677.95** | **22677.95** | **22677.95** |
| **77** | **PG(50:7)+NH4** | **44296.67** | **44296.67** | **44296.67** | **233991.6** | **246500** | **221483.3** | **44296.67** | **44296.67** | **44296.67** |
| **78** | **PI(32:2)+H** | **187614.3** | **187614.3** | **187614.3** | **938071.4** | **958617.1** | **979162.9** | **187614.3** | **187614.3** | **187614.3** |
| **79** | **PI(40:5)+H** | **23176.28** | **23176.28** | **23176.28** | **23176.28** | **23176.28** | **23176.28** | **123172.2** | **115881.4** | **119526.8** |
| **80** | **PI(38:3)+NH4** | **43595.38** | **43595.38** | **43595.38** | **217976.9** | **253295.7** | **235636.3** | **43595.38** | **43595.38** | **43595.38** |
| **81** | **PI(40:7)+NH4** | **219725.3** | **257993** | **296260.6** | **43945.06** | **43945.06** | **43945.06** | **43945.06** | **43945.06** | **43945.06** |
| **82** | **PI(40:8)+H** | **27987.77** | **31445.39** | **27757.3** | **5551.46** | **5551.46** | **5551.46** | **5551.46** | **5551.46** | **5551.46** |
| **83** | **PI(40:8)+NH4** | **232287.2** | **208945.8** | **236534.1** | **41789.16** | **41789.16** | **41789.16** | **41789.16** | **41789.16** | **41789.16** |
| **84** | **PI(40:9)+NH4** | **4777.67** | **4777.67** | **4777.67** | **79443.85** | **72482.99** | **62154.67** | **23888.35** | **26875.19** | **27684.99** |
| **85** | **PI(36:5)+H** | **355161.6** | **271866.5** | **316891.9** | **346046.6** | **359973** | **373899.4** | **331269.1** | **258322.2** | **404216** |
| **86** | **PI(40:5)+NH4** | **715089.5** | **269416.2** | **492252.9** | **898321.5** | **885972.8** | **910670.2** | **935051.7** | **799582.2** | **1070521** |
| **87** | **PS(38:5)+H** | **669591.6** | **1183927** | **926759.2** | **133918.3** | **133918.3** | **133918.3** | **3109732** | **8970914** | **6040323** |
| **88** | **PS(38:1)+H** | **123734.6** | **123734.6** | **123734.6** | **618673.1** | **695558.4** | **657115.8** | **123734.6** | **123734.6** | **123734.6** |
| **89** | **PS(40:2)+H** | **1670655** | **1703957** | **1737260** | **406830.7** | **453111** | **486475** | **499329.6** | **478008.9** | **515314.6** |
| **90** | **PS(40:4)+H** | **664679.8** | **658586.4** | **661633.1** | **131717.3** | **131717.3** | **131717.3** | **131717.3** | **131717.3** | **131717.3** |
| **91** | **PS(42:3)+H** | **211984.4** | **193311.1** | **230657.8** | **38662.22** | **38662.22** | **38662.22** | **38662.22** | **38662.22** | **38662.22** |
| **92** | **PS(36:4)+H** | **1037000** | **1200701** | **873299.5** | **72864.43** | **72864.43** | **72864.43** | **916331.4** | **640326.8** | **364322.2** |
| **93** | **PS(40:6)+H** | **31276.53** | **31276.53** | **31276.53** | **31276.53** | **31276.53** | **31276.53** | **262116.8** | **367850.9** | **156382.7** |
| **94** | **SPH(d16:0)+H** | **81400000** | **67900000** | **73500000** | **47300000** | **46800000** | **44100000** | **80200000** | **61800000** | **62300000** |
| **95** | **TG(46:4)+NH4** | **1705271** | **2021987** | **1863629** | **22900000** | **23700000** | **19500000** | **341054.1** | **341054.1** | **341054.1** |
| **96** | **TG(48:6)+NH4** | **3244585** | **3052225** | **2047994** | **409598.8** | **409598.8** | **409598.8** | **409598.8** | **409598.8** | **409598.8** |
| **97** | **TG(46:1)+NH4** | **1.19E+08** | **1.1E+08** | **1.15E+08** | **4490333** | **4282177** | **4074020** | **814804** | **814804** | **814804** |
| **98** | **TG(52:10)+NH4** | **95021.42** | **95021.42** | **95021.42** | **6314542** | **5516695** | **4718848** | **475107.1** | **556181.2** | **505975.5** |
| **99** | **TG(50:0)+NH4** | **323172.1** | **323172.1** | **323172.1** | **2717932** | **1720047** | **1615860** | **323172.1** | **323172.1** | **323172.1** |
| **100** | **TG(52:0)+NH4** | **274255.2** | **274255.2** | **274255.2** | **1397369** | **1371276** | **1423462** | **274255.2** | **274255.2** | **274255.2** |
| **101** | **TG(54:2)+NH4** | **2180000** | **2180000** | **2180000** | **10900000** | **11600000** | **12300000** | **11300000** | **10900000** | **11100000** |
| **102** | **TG(58:3)+NH4** | **1679696** | **1679696** | **1679696** | **1679696** | **1679696** | **1679696** | **8766188** | **9133894** | **8398482** |
| **103** | **TG(68:11)+NH4** | **35699.8** | **35699.8** | **35699.8** | **35699.8** | **35699.8** | **35699.8** | **229078.2** | **279657.4** | **178499** |
| **104** | **TG(50:3)+NH4** | **69700000** | **68600000** | **67400000** | **13480000** | **13480000** | **13480000** | **13480000** | **13480000** | **13480000** |
| **105** | **TG(50:4)+NH4** | **3420000** | **3420000** | **3420000** | **17100000** | **19300000** | **21600000** | **3420000** | **3420000** | **3420000** |
| **106** | **TG(56:6)+NH4** | **1.09E+08** | **1.13E+08** | **1.16E+08** | **2.18E+07** | **2.18E+07** | **2.18E+07** | **2.18E+07** | **2.18E+07** | **2.18E+07** |
| **107** | **TG(66:7)+NH4** | **24741.7** | **24741.7** | **24741.7** | **306330.5** | **277976** | **334685** | **123708.5** | **146192.6** | **168676.6** |
| **108** | **TG(50:10)+NH4** | **115361.5** | **115361.5** | **115361.5** | **576807.3** | **650986.9** | **725166.5** | **115361.5** | **115361.5** | **115361.5** |
| **109** | **TG(52:11)+NH4** | **1084782** | **1084782** | **1084782** | **5885550** | **5423908** | **5973314** | **1084782** | **1084782** | **1084782** |
| **110** | **TG(56:2)+NH4** | **6795892** | **7056913** | **7317934** | **1359178** | **1359178** | **1359178** | **1359178** | **1359178** | **1359178** |
| **111** | **TG(60:3)+NH4** | **192680.1** | **192680.1** | **192680.1** | **192680.1** | **192680.1** | **192680.1** | **1018652** | **991026** | **963400.3** |
| **112** | **TG(58:8)+NH4** | **1.31E+07** | **1.31E+07** | **1.31E+07** | **1.31E+07** | **1.31E+07** | **1.31E+07** | **1.56E+08** | **6.55E+07** | **7.63E+07** |
| **113** | **TG(56:8)+NH4** | **65800000** | **66200000** | **66600000** | **9400000** | **9400000** | **9400000** | **70000000** | **47000000** | **93000000** |
| **114** | **TG(62:6)+NH4** | **578469.8** | **659238.1** | **740006.4** | **2722629** | **2612071** | **2833186** | **115694** | **115694** | **115694** |
| **115** | **TG(62:8)+NH4** | **397260.8** | **397260.8** | **397260.8** | **2317867** | **1986304** | **2649429** | **11800000** | **9280000** | **6777018** |
| **116** | **TG(62:10)+NH4** | **3380000** | **3380000** | **3380000** | **3380000** | **3380000** | **3380000** | **16900000** | **17200000** | **17400000** |
| **117** | **TG(64:10)+NH4** | **1036918** | **1060304** | **1083690** | **207383.6** | **207383.6** | **207383.6** | **207383.6** | **207383.6** | **207383.6** |
| **118** | **TG(56:11)+NH4** | **1201647** | **1201647** | **1201647** | **1201647** | **1201647** | **1201647** | **9026674** | **6008236** | **7517455** |
| **119** | **TG(58:14)+NH4** | **333856.4** | **333856.4** | **333856.4** | **333856.4** | **333856.4** | **333856.4** | **2016012** | **1669282** | **2017797** |
| **120** | **TG(60:15)+NH4** | **62177.94** | **81871.06** | **85064.63** | **19400000** | **18200000** | **20300000** | **1757692** | **2233303** | **1995497** |
| **121** | **TG(62:15)+NH4** | **378217.5** | **314304.8** | **442130.2** | **62860.96** | **62860.96** | **62860.96** | **62860.96** | **62860.96** | **62860.96** |
| **122** | **TG(62:16)+NH4** | **800505** | **800505** | **800505** | **4006733** | **4253748** | **4002525** | **800505** | **800505** | **800505** |
| **123** | **TG(64:15)+NH4** | **768436.7** | **834693.6** | **801565.2** | **153687.3** | **153687.3** | **153687.3** | **153687.3** | **153687.3** | **153687.3** |
| **124** | **TG(68:9)+NH4** | **31087.26** | **31087.26** | **31087.26** | **31087.26** | **31087.26** | **31087.26** | **162595.1** | **155436.3** | **159015.7** |
| **125** | **TG(66:11)+NH4** | **312438.4** | **259012.2** | **283802.2** | **51802.44** | **51802.44** | **51802.44** | **51802.44** | **51802.44** | **51802.44** |
| **126** | **TG(66:15)+NH4** | **501039** | **503259.9** | **487826.9** | **47235.17** | **47235.17** | **47235.17** | **246223** | **241199.4** | **236175.9** |
| **127** | **TG(68:8)+NH4** | **10480.4** | **10480.4** | **10480.4** | **52401.99** | **160529** | **142769.2** | **10480.4** | **10480.4** | **10480.4** |
| **128** | **TG(56:14)+NH4** | **430484** | **430484** | **430484** | **2640827** | **2300352** | **2152420** | **430484** | **430484** | **430484** |
| **129** | **TG(58:15)+NH4** | **75787.32** | **75787.32** | **75787.32** | **425604.8** | **378936.6** | **402270.7** | **75787.32** | **75787.32** | **75787.32** |

**Table S4 :** List of normalized area of lipids in hemolymph of female and male abalone, *Haliotis discus hannai*

| **No.** | **Lipid name** | **female** | | | **male** | | |
| --- | --- | --- | --- | --- | --- | --- | --- |
|  |  | **female1** | **female2** | **female3** | **male1** | **male2** | **male3** |
| **1** | **Ch+H-H2O** | **480809.8** | **253965.1** | **707654.6** | **363810.5** | **383886.7** | **343734.2** |
| **2** | **DG(34:0)+NH4** | **36724604** | **19637786** | **31829680** | **58475721** | **75415253** | **56035611** |
| **3** | **DG(34:1)+NH4** | **716196.5** | **341865.1** | **349394.1** | **68373.02** | **68373.02** | **68373.02** |
| **4** | **MG(14:0)+H** | **89668.38** | **89668.38** | **89668.38** | **448341.9** | **509068.5** | **478705.2** |
| **5** | **MG(16:0)+H** | **3937485** | **3114024** | **5314055** | **2.50E+07** | **33400000** | **29200000** |
| **6** | **PC(32:0)+H** | **1007534** | **404770.7** | **706152.5** | **703343.4** | **574821.1** | **446298.7** |
| **7** | **PC(32:1)+H** | **353287.7** | **353287.7** | **353287.7** | **1766438** | **1905117** | **1835778** |
| **8** | **PC(34:2)+H** | **1063765** | **652117.9** | **636535.2** | **477033.5** | **524626.5** | **455202.4** |
| **9** | **PC(34:3)+H** | **9347.91** | **9347.91** | **9347.91** | **46739.55** | **82006.59** | **117273.6** |
| **10** | **PC(34:4)+H** | **724156.6** | **610860.8** | **667508.7** | **625291.2** | **517314.4** | **571302.8** |
| **11** | **PC(34:5)+H** | **129134.3** | **132847.8** | **136561.3** | **108810.2** | **103506.3** | **114114.1** |
| **12** | **PC(38:2)+H** | **541001.5** | **302895.3** | **421948.4** | **271532.6** | **564669.4** | **418101** |
| **13** | **PC(38:5)+H** | **2956509** | **2843532** | **3069487** | **2161877** | **1834790** | **1507704** |
| **14** | **PE(40:3)+H** | **10329.96** | **10329.96** | **10329.96** | **55668.62** | **59687.45** | **51649.8** |
| **15** | **PS(40:2)+H** | **4650.248** | **4650.248** | **4650.248** | **23251.24** | **46416.56** | **34833.9** |
| **16** | **SPH(d16:0)+H** | **1.95E+08** | **1.52E+08** | **1.94E+08** | **2.07E+08** | **2.27E+08** | **1.86E+08** |
| **17** | **TG(46:1)+NH4** | **25397.67** | **25397.67** | **25397.67** | **133421** | **139853.7** | **126988.4** |
| **18** | **TG(50:0)+NH4** | **527812.1** | **590376.2** | **465248.1** | **8479958** | **8197448** | **8261517** |
| **19** | **TG(50:1)+NH4** | **61559.24** | **61559.24** | **61559.24** | **361620.2** | **307796.2** | **334708.2** |
| **20** | **TG(54:0)+NH4** | **567887.3** | **597567.5** | **538207.1** | **107641.4** | **107641.4** | **107641.4** |
| **21** | **TG(56:1)+NH4** | **103418.7** | **117463.6** | **89373.79** | **17874.76** | **17874.76** | **17874.76** |

**Figure S1 :** Total ion current (TIC) chromatogram of the tissue samples: (a) Swimming veliger larvae, (b) Juvenile, (c) Mature. All the samples were analyzed triplicate(ⅰ), (ⅱ), (ⅲ).

(a) Swimming veliger larvae

(ⅰ)


(ⅱ)

(ⅲ)

(b) Juvenile

(ⅰ)

(ⅱ)

(ⅲ)

(c) Mature

(ⅰ)

(ⅱ)

(ⅲ)

**Figure S2 :** Total ion current (TIC) chromatogramof the hemolymph samples: (a) Female, (b) Male. All the samples were analyzed triplicate(ⅰ), (ⅱ), (ⅲ).

(a) Female

(ⅰ)

(ⅱ)

(ⅲ)

(b) Male

(ⅰ)

(ⅱ)

(ⅲ)

**Figure S3 :** PCA score plots of (a) tissues in three different developmental stages, (b) female and male hemolymph

(a) (b)

**
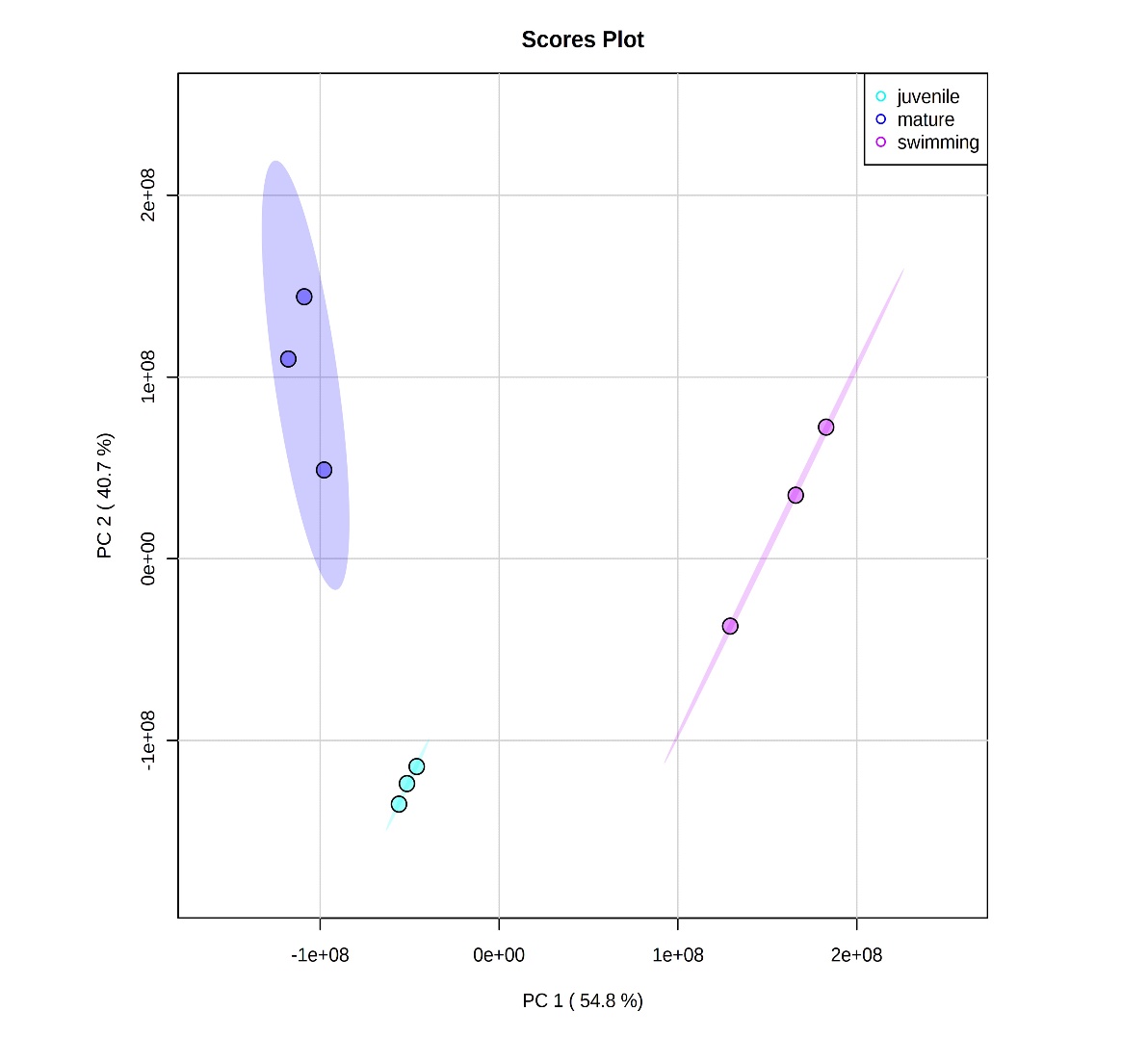

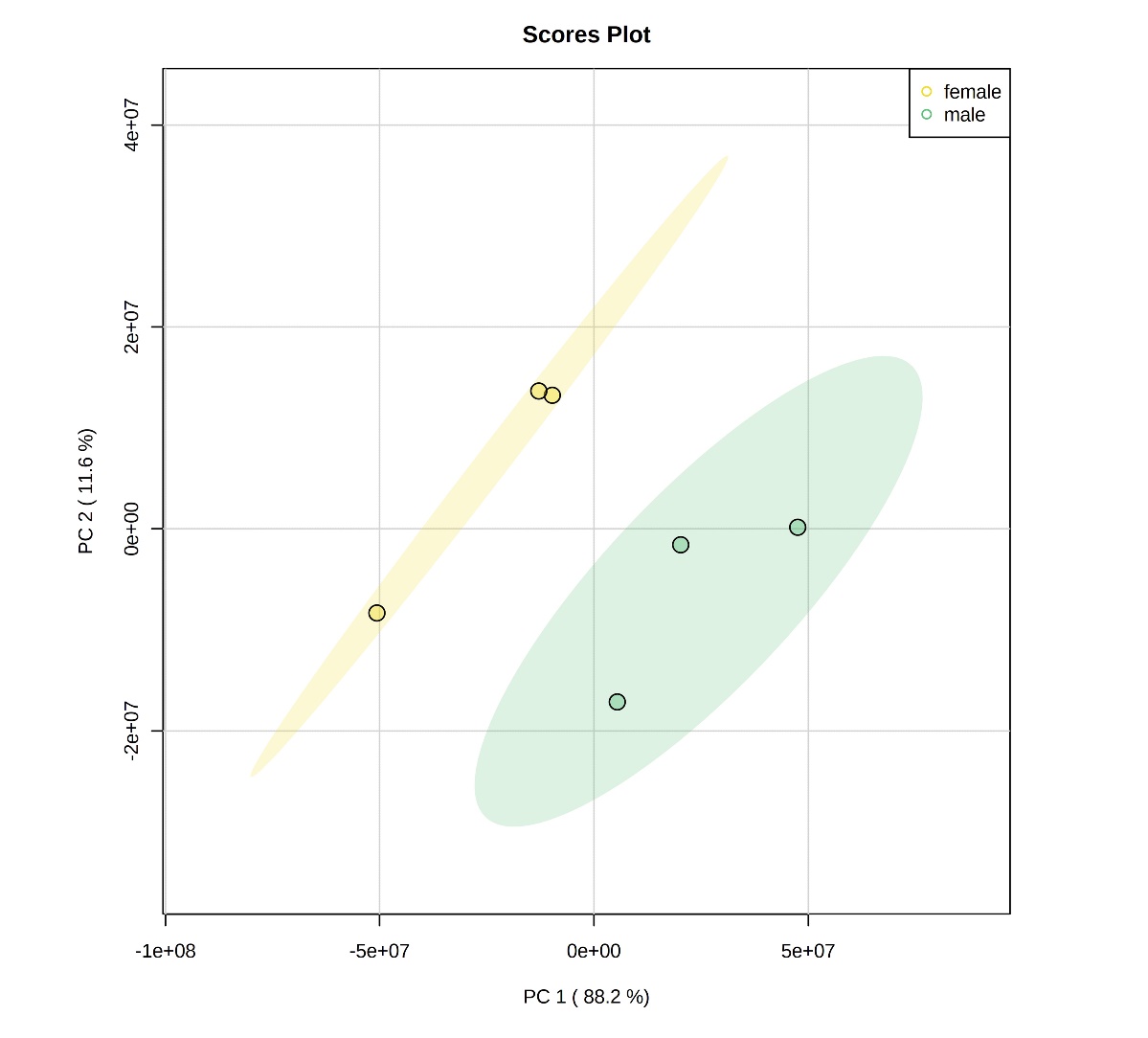
**

**Figure S4 :** The detailed heatmap of abalone tissue


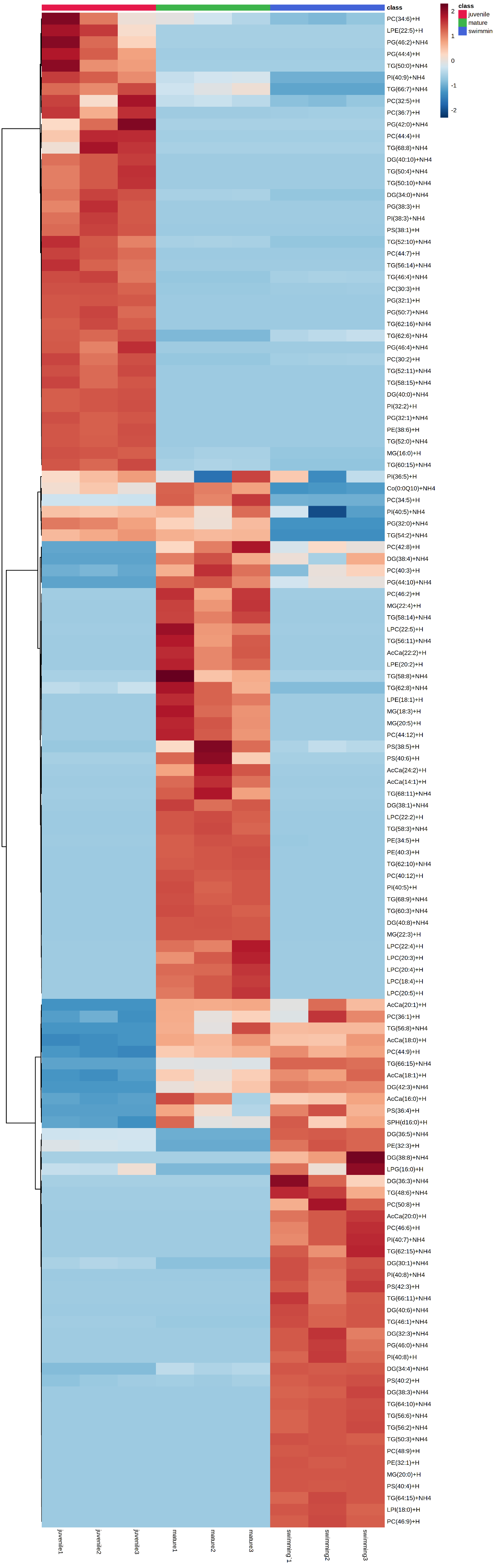


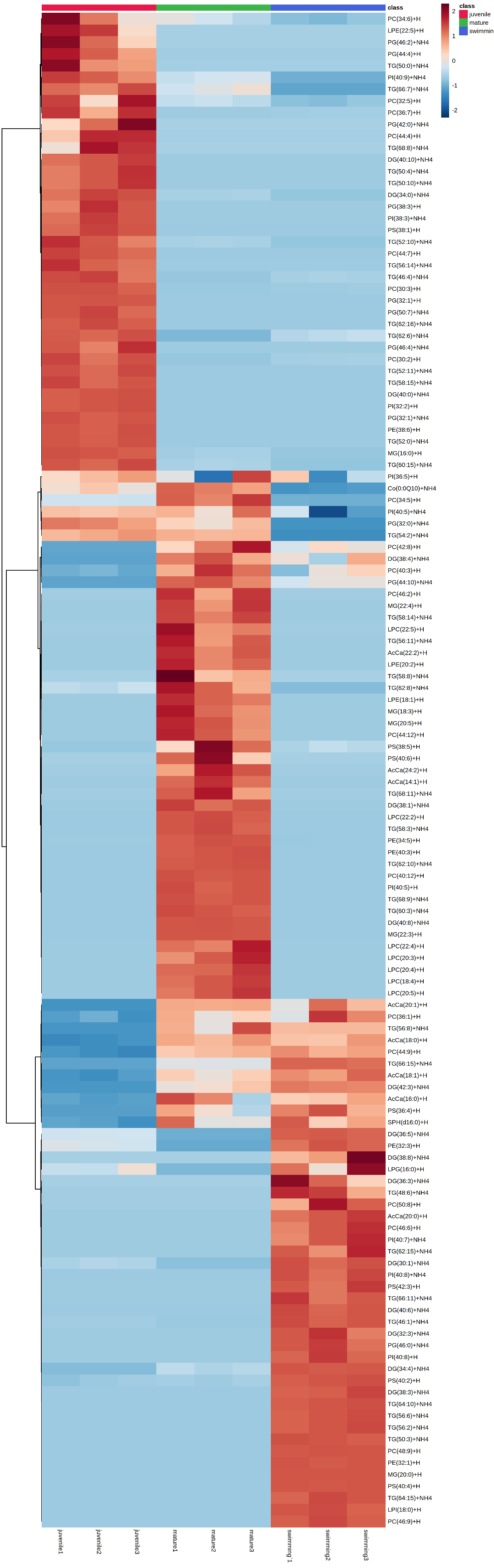

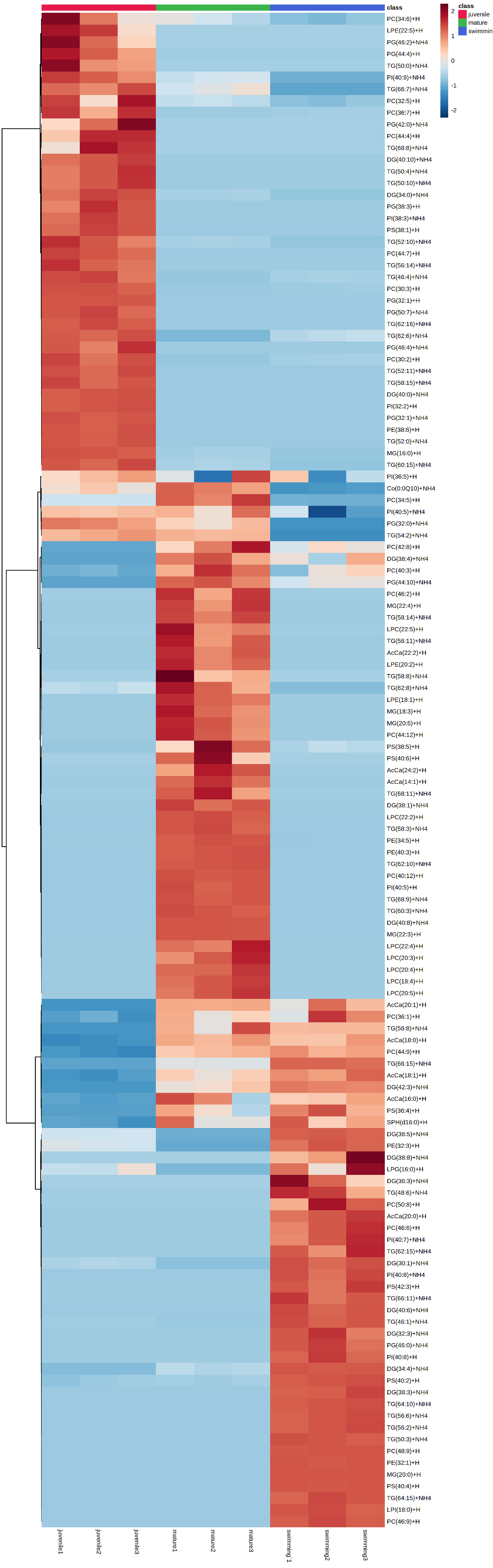

Supplement: Supplementary Materials — Table S1: List of identified lipids in tissue of abalone, Haliotis discus hannai at different developmental stages (swimming veliger larvae, juvenile, mature). Table S2: List of identified lipids in hemolymph of female and male abalone, Haliotis discus hannai. Table S3: List of the normalized area of lipids in tissue of abalone, Haliotis discus hannai at different developmental stages (swimming veliger larvae, juvenile, mature). Table S4: List of normalized area of lipids in hemolymph of female and male abalone, Haliotis discus hannai. Figure S1: Total ion current (TIC) chromatogram of the tissue samples: (a) Swimming veliger larvae, (b) Juvenile, (c) Mature. Figure S2: Total ion current (TIC) chromatogram of the hemolymph samples: (a) Female, (b) Male. Figure S3: PCA score plots of (a) tissues in three different developmental stages, (b) female and male hemolymph. Figure S4: The detailed heatmap of abalone tissue. [file 5822562.f1.docx]
